# Make or break - PEDS1 and AGMO orchestrate ether lipid homeostasis in human adipocytes and are associated with blood lipid profiles

**DOI:** 10.1186/s12967-026-07728-8

**Published:** 2026-01-24

**Authors:** Sabrina Sailer, Tina Deutinger, Susanne Lobenwein, Katharina Lackner, Stephan Geley, Georg Golderer, Ernst R. Werner, Markus A. Keller, Christian Ploner, Katrin Watschinger

**Affiliations:** 1 Institute of Human Genetics, Medical University of Innsbruck, Innsbruck, 6020 Austria; 2 Department of Plastic, Reconstructive and Aesthetic Surgery, Medical University of Innsbruck, Innsbruck, 6020 Austria; 3 Institute of Molecular Pathophysiology, Biocenter, Medical University of Innsbruck, Innsbruck, 6020 Austria; 4 Institute of Molecular Biochemistry, Biocenter, Medical University of Innsbruck, Innsbruck, 6020 Austria

**Keywords:** Ether lipid metabolism, Adipose tissue, Lipidomics, Lipoprotein particles, Plasmanylethanolamine desaturase, Alkylglycerol monooxygenase

## Abstract

**Background:**

Obesity and its associated sequelae have become a major global health concern. While ester-linked lipids are well-established regulators of adipocyte function and energy storage. Emerging evidence from cohort studies suggests that ether-linked lipid levels are also altered during the development of acquired obesity. The genes of two extra-peroxisomal enzymes of ether lipid metabolism, alkylglycerol monooxygenase (*AGMO*) and plasmanylethanolamine desaturase (*PEDS1*), have recently been identified, but their physiological roles in humans remain poorly understood.

**Methods:**

We conducted a monocentric, cross-sectional study analyzing subcutaneous adipose tissue from 30 patients undergoing abdominoplasty. To dissect the function of AGMO and PEDS1, we combined in vitro knockdown experiments in adipocyte-derived stem cells with pulse-chase tracing of a labeled ether lipid precursor. Untargeted lipidomics using liquid chromatography–tandem mass spectrometry was applied to in vivo differentiated adipocytes to assess the impact of AGMO activity on phospholipid composition. To explore a systemic relevance for AGMO and PEDS1 in humans, enzyme activity and gene expression data were correlated with blood lipid and metabolic parameters.

**Results:**

We demonstrate that AGMO and PEDS1 are critical regulators of ether lipid subclass balance during adipocyte differentiation. Both enzymes maintained plasmanyl- and plasmenylphospholipid homeostasis without altering total ether lipid levels, indicating dynamic remodeling rather than a metabolic bottleneck. In vivo, AGMO activity reshaped the molecular phospholipid composition of primary adipocytes. Importantly, expression and activity of AGMO and PEDS1 correlated with circulating cholesterol, triglycerides, and lipoprotein particle levels, linking adipose ether lipid metabolism to systemic lipid regulation.

**Conclusion:**

This is the first human study highlighting AGMO and PEDS1 as key determinants of adipose ether lipid remodeling with systemic metabolic relevance. By connecting adipocyte ether lipid metabolism to circulating lipid profiles, our findings point to AGMO and PEDS1 as promising candidates for further investigation in the context of obesity and metabolic syndrome, and may warrant further exploration as potential contributors to mitigating metabolic dysregulation.

**Supplementary Information:**

The online version contains supplementary material available at 10.1186/s12967-026-07728-8.

## Background

Adipose tissue is an important endocrine organ comprising two main subtypes: brown adipose tissue (BAT) and white adipose tissue (WAT). In addition to its role in energy storage, thermogenesis, and the regulation of free fatty acid levels in the blood, adipose tissue is also a source of signaling peptides and cytokines, collectively known as adipokines [1]. These secreted factors regulate a number of physiological conditions, including appetite, energy homeostasis, inflammation and insulin sensitivity. Adipose conversion, commitment and differentiation of a non-specialized cell into a mature adipocyte is a highly intricate mechanism comprising multiple phases that regulate over 14,000 mRNA transcripts [2, 3]. The prevalence of obesity and its associated comorbidities has reached a critical level, posing a significant public health concern not only in developed countries [4]. As reported by the NCD Risk Factor Collaboration (NCD-RisC), the global population is gaining approximately 1.5 kg in weight each decade [5]. A number of lipidomic studies have demonstrated that the quantity and occurrence of ether-linked lipid species are altered in several tissues in response to weight gain [6–8].

The synthesis of ether lipids commences in peroxisomes and subsequently progresses in the endoplasmic reticulum and they can be classified into two distinct subclasses, the plasmanyl- and plasmenyl-ether lipids. The fatty alcohol present at the *sn*-1 position of the glycerol backbone of ether lipids is derived through the reduction of fatty acids by fatty acyl-CoA reductase 1/2 (FAR1/FAR2) [9]. It is then attached by an alkyl ether bond (in plasmanyl ether lipids) that can undergo desaturation to form a vinyl ether bond (plasmenyl ether lipids or plasmalogens). Human ether lipid deficiency disorders are caused by defects in peroxisomal assembly (involving 12 *PEX* genes) or mutations in peroxisomal enzymes [10]. Knockout mouse models mimicking global ether lipid deficiency have provided insights into these conditions, though with milder phenotypes than in humans [11]. While the biosynthetic roles of peroxisomal enzymes in ether lipid metabolism are well established, the functions of extraperoxisomal enzymes remain comparatively less well characterized. One such enzyme is plasmanylethanolamine desaturase (PEDS1, E.C. 1.14.99.19), which catalyzes the formation of the characteristic vinyl ether bond in plasmalogens.

The gene encoding PEDS1 was only recently identified between 2019 and 2021 by three independent groups [12–14], and the enzymatic process has been described in detail elsewhere [15]. A *Peds1*-deficient knockout mouse model exhibited a lack of plasmalogens and delayed growth and weight gain following weaning [13]. The ether bond confers unique biochemical properties to these lipids, including stability of plasmanyl lipids to acid-base treatment, and physico-chemical properties of plasmalogens that influence the rigidity of cell membranes. In contrast to PEDS1, alkylglycerol monooxygenase (AGMO, EC 1.14.16.5) is the only known enzyme to oxidise and, thereby, degrade plasmanyl lipids [16]. An *Agmo* knockout mouse model was established in our laboratory, yet no pathologic phenotype was observed under standard germ-free housing conditions [17]. A potential physiological role for AGMO in human pathologies such as type 2 diabetes and neurological disorders has gradually emerged (reviewed in [18]). Genome-wide association studies have linked mutations in the AGMO gene or its intergenic region to key metabolic and pathological processes, including energy homeostasis in obesity [19], glucose homeostasis [19, 20] and cancer [21]. Despite growing evidence that ether lipid homeostasis and AGMO are key players in systemic energy balance and obesity, their specific role in adipocyte biology remains poorly defined. This is partly due to technical challenges in distinguishing ether lipid species marked by O- (plasmanyl) or P- (plasmenyl) bonds [22] and because several key genes involved in their metabolism have only recently been identified [23]. Recent advances in lipidomics now enable more accurate lipid profiling [24]. In vitro adipogenesis studies have shown increased levels of neutral ether lipids, such as monoalkyl diacylglycerol (TG(O)) [25, 26], while plasmalogen phospholipids (PL) — comprising ~ 20% of total PL mass — accumulate with adipocyte expansion to preserve membrane integrity [27].

This study aimed to elucidate the physiological roles of AGMO and PEDS1 in human adipocyte biology and to explore ether lipid metabolism during adipocyte differentiation in primary cells. We demonstrate for the first time that both enzymes are expressed and active throughout in vitro adipogenesis of human adipose-derived stem cells (ASC). Notably, we identified novel correlations between AGMO activity, PEDS1 expression, and basic blood parameters in in vivo differentiated adipocytes. Moreover, untargeted lipidomics revealed AGMO-dependent shifts in phospholipid composition. The findings presented herein underscore the significance of coordinated ether lipid turnover in human adipose tissue and the correlation between circulating blood parameters and this metabolic process.

## Materials and methods

The following is a brief description of the **KEY methods**. More detailed methodologies can be found in Supplementary Material 1.

### Study

#### Study population

We recruited 30 patients, both male and female, aged 18 to 68 years, who underwent abdominoplasty surgery at the Department of Plastic, Reconstructive and Aesthetic Surgery (Medical University of Innsbruck, Austria) between January 2019 and January 2021. Only written informed consent from competent patients was permitted and no further risk was inflicted on the patient. All patients undergoing elective abdominoplasty were eligible. Participants were only excluded when informed consent was withdrawn. For this study, all samples and clinical parameters were received in a pseudonymized manner.

For correlation studies of AGMO in human tissue samples, a sample size estimation has been calculated with the R package ‘pwr’. For this, an approximate sample size of 28 patients was calculated at an assumed linear correlation coefficient (r) of 0.5, α = 0.05, power (1-β) = 0.8 and two-tailed significance testing.

#### Study outcomes

The primary outcomes focused on the characterization of the key metabolic enzymes AGMO and PEDS1 in human adipose-derived stem cells (ASC) differentiation and primary in vivo differentiated adipocytes. Additionally, correlation analyses were performed with basic blood parameters (mainly focusing on lipoprotein particles and lipid-related parameters such as cholesterol and triglycerides) routinely measured in standard clinical diagnostics, aiming to explore associations between enzyme expression or activity and systemic metabolic profiles.

#### Study design

This monocentric study was conducted as part of translational research to better understand ether lipid metabolism in human adipose tissue biology. By analyzing human-derived samples, the study aimed to identify depot- and cell type–specific features relevant to metabolic regulation and clinical parameters.

#### Bias and limitations

To minimize selection bias, all available human adipose tissue samples were included without selection based on clinical outcomes. However, as a monocentric retrospective study, inherent limitations such as non-random sampling and potential clinical heterogeneity of the donor population may introduce bias. Data collection relied on existing clinical records and sample availability; no imputation was performed for missing data, and analyses were restricted to available cases (complete-case analysis). Potential confounders such as age, sex, and BMI were considered during analysis where appropriate, but residual confounding cannot be fully excluded.

### Isolation of human adipose tissue-derived stem cells and in vivo differentiated adipocytes

Subcutaneous fat tissue was retrieved from patients of elective abdominoplastic surgery, which was approved by the Ethics Committee of the Medical University of Innsbruck (AN2014-0244 341/4.5 388/5.5). Written informed consent was obtained from all donors and the methods were carried out in accordance with the approved guidelines. Human ASC and in vivo differentiated adipocytes were isolated according to Morandi et al. [28].

### Adipocyte differentiation of human ASC and staining of lipid droplets

After isolation, ASC were plated at an initial density of 6 × 10^5^ cells / well of a 6-well plate or 1.2 × 10^5^ cells / well of a 24-well plate and the following day, adipocyte differentiation was induced by adding an adipogenic induction medium according to [28] with some minor modifications (see Supplemental Material and Methods). Lipid droplets of in vitro differentiated adipocytes were stained with Bodipy™ 493/503 (Fisher Scientific, Vienna, Austria) and nuclei were stained with Hoechst (H 33342, Merck, Darmstadt, Germany).

### 3T3-L1 differentiation and knockdown of AGMO

Lentiviral knockdown of AGMO and subsequent adipocyte differentiation of 3T3-L1 preadipocytes were performed as previously described [26]. A second knockdown line targeting positions 506–524 of the murine *Agmo* locus (GenBank accession no. NM_178767.5) was added to the experiments conducted in this study.

### RNA isolation and gene expression analysis

Total RNA from undifferentiated and differentiated ASC cells was prepared using the Monarch Total RNA Miniprep kit according to the manufacturer’s protocol (NEB, Frankfurt, Germany). Total RNA from in vivo differentiated human adipocytes was isolated using the RNeasy Lipid Tissue Mini kit (Qiagen, Hilden, Germany) according to the manufacturer’s protocol with a minor modification (see Supplemental Material and Methods). Transcription into cDNA was performed for all RNAs using the M-MLV reverse transcriptase (RNase H Minus, Point Mutant; Promega, Mannheim, Germany) and random hexamer primers (Microsynth, Balgach, Switzerland). For qPCR, the TaqMan assay technology using Brilliant III Ultra-Fast QPCR Master Mix (Agilent Technologies, Vienna, Austria) and the Mx3005P qPCR system (Agilent) were used. Taqman probes were labelled with FAM (5’) and TAMRA (3’). Primer and Taqman probe sequences are listed in Supplemental Table S1.

### Enzyme activity assays

AGMO enzyme activity was measured as described in a previous work [29] with the following modifications: Homogenates of differentiated and undifferentiated ASC were not centrifuged and a protein concentration of ≥ 1 mg/ml was used to measure the enzymatic activity. Furthermore, fatty aldehyde dehydrogenase, essential for full conversion of the fatty aldehyde to the fatty acid, was added in its recombinant form to the assay mixture [30]. PEDS1 enzyme activity was measured as described in a previous work [31].

### Lipid extraction

ASC were supplemented with 5 µM 1-*O*-pyrenedecyl-*sn*-glycerol [29, 32] for 24 h and afterwards, cells were trypsinized, centrifuged and dry cell pellets were snap frozen and stored at -80 °C until analysis. Lipids from frozen cell pellets were extracted according to [31] and analyzed by reversed-phase HPLC. To further quantify formation of ether-linked neutral lipids as well as phosphatidylethanolamine (PE) and phosphatidylcholine (PC), crude lipid extracts were processed by solid phase extraction according to [33]. Lipids from 200 µg net weight isolated primary adipocytes were extracted using the Folch method [34] with the addition of 0.01% butylated hydroxytoluene (BHT, Sigma, Vienna, Austria) to the chloroform: methanol mixture containing 5 µl of internal Splash^®^ Lipidomix^®^ mass spec standard per sample (Avanti Polar Lipids, Alabaster, AL, USA). From the same extract, a second extraction with hexane: ethanol: H_2_O was performed to remove neutral lipids and is described in detail in [35]. Derivatization of plasmalogens using dansylhydrazine (Sigma, 0.45 mg/ml in acetonitrile/2 M aqueous HCl (930:70 v/v)) was performed as previously described [31]. Extracts were immediately measured within 24 h after reconstitution. Acylcarnitines were extracted from 20 µl in vivo differentiated adipocyte cell suspension using 3:1 (v/v) acetonitrile: methanol. Internal acylcarnitine standard NSK-B-1 and NSK-B-G1-1 were purchased from Cambridge Isotope Laboratories, Inc. (Tewksbury, MA, USA). Additionally, malonyl-L-carnitine-(N-methyl-d3), hexanoyl-L-carnitine-(N-methyl-d3) and decanoyl-L-carnitine-(N-methyl-d3) (Sigma) were supplemented to the internal standard mixture. In brief, samples were processed by beat beating on a cryomill for 2.5 min at 20 Hz, sonicating for 5 min and centrifuging for 10 min at 31,000 g. Supernatant was transferred to a glass vial, evaporated under a stream of nitrogen at 37 °C and reconstituted in 100 µl 3:1 acetonitrile: methanol (v/v). Extracts were immediately measured after reconstitution.

### Lentiviral transduction of human ASC

Generation of pHR-SFFV-PURO plasmid was described previously [36]. Cloning of short hairpin (sh) RNAs into the lentiviral transduction plasmid pHR-DEST-SFFV-Puro was performed according to [37] and contained the shRNA-encoding oligonucleotides of either human AGMO ((i) 343–361 for sh*AGMO*343 or (ii) 1699–1717 for sh*AGMO*1699 (GeneBank accession no. NM_001004320.2)) or the human *PEDS1* gene ((iii) 928–946 for sh*PEDS1*928 or (iv) 940–958 for sh*PEDS1*940 (GeneBank accession no. NM_199129.3)).

### Mass spectrometric analysis of phospholipids and acylcarnitines

For phospholipid analysis, an Agilent InfinityLab Poroshell 2.7 μm (120 EC-C8; 2.1 × 100 mm) (Agilent) was used. A Vanquish UHPLC system (Thermo Fisher Scientific, Waltham, MA, USA) was coupled to a timsTof Pro ion mobility mass spectrometer equipped with a VIP-HESI source (Bruker, Bremen, Germany). A detailed description of mass spectrometry parameters can be found in Supplemental Table S2. In brief, data-dependent acquisition with parallel accumulation-serial fragmentation (DDA-PASEF) was used and operated in negative ion mode. Untargeted phospholipid analysis was done with Metaboscape2021b (Bruker). Annotated lipids were verified by linear regression analysis of published retention times from Lange et al. [35]. To confirm plasmalogen annotations, samples were compared with lipid extracts from *Peds1* knockout mice that were measured in parallel. Acylcarnitines were analyzed by hydrophilic interaction liquid chromatography by injecting 5 µl of the extract onto an Acquity UPLC^®^ BEH HILIC 1.7 μm column (2.1 × 100 mm) (Waters, Milford, MA, USA).

### Statistics

If not indicated otherwise, data are presented as the mean ± standard error of the mean (SEM). Boxplots show the median ± interquartile range (IQR) with the whiskers spreading from minimum to maximum. Gaussian distributed data were compared by Student’s t-test or by 2-way ANOVA. For correlation analysis of nonparametric data, Spearman’s Rank Correlation was computed in R Studio (2023.12.0). Data was analyzed in GraphPad Prism 5.01 (GraphPad Software Inc., San Diego, CA, USA) or Microsoft Excel 2010 (Microsoft Corporation, Redmond, WA, USA) was used. P values < 0.05 were considered as statistically significant. * *P <* 0.05, ** *P <* 0.01, *** *P <* 0.001 and **** *P <* 0.0001. For multiple comparisons, P values were (L)FDR adjusted.

## Results

### Characterization of post-peroxisomal ether lipid metabolism during human adipocyte differentiation

**Fig. 1:**
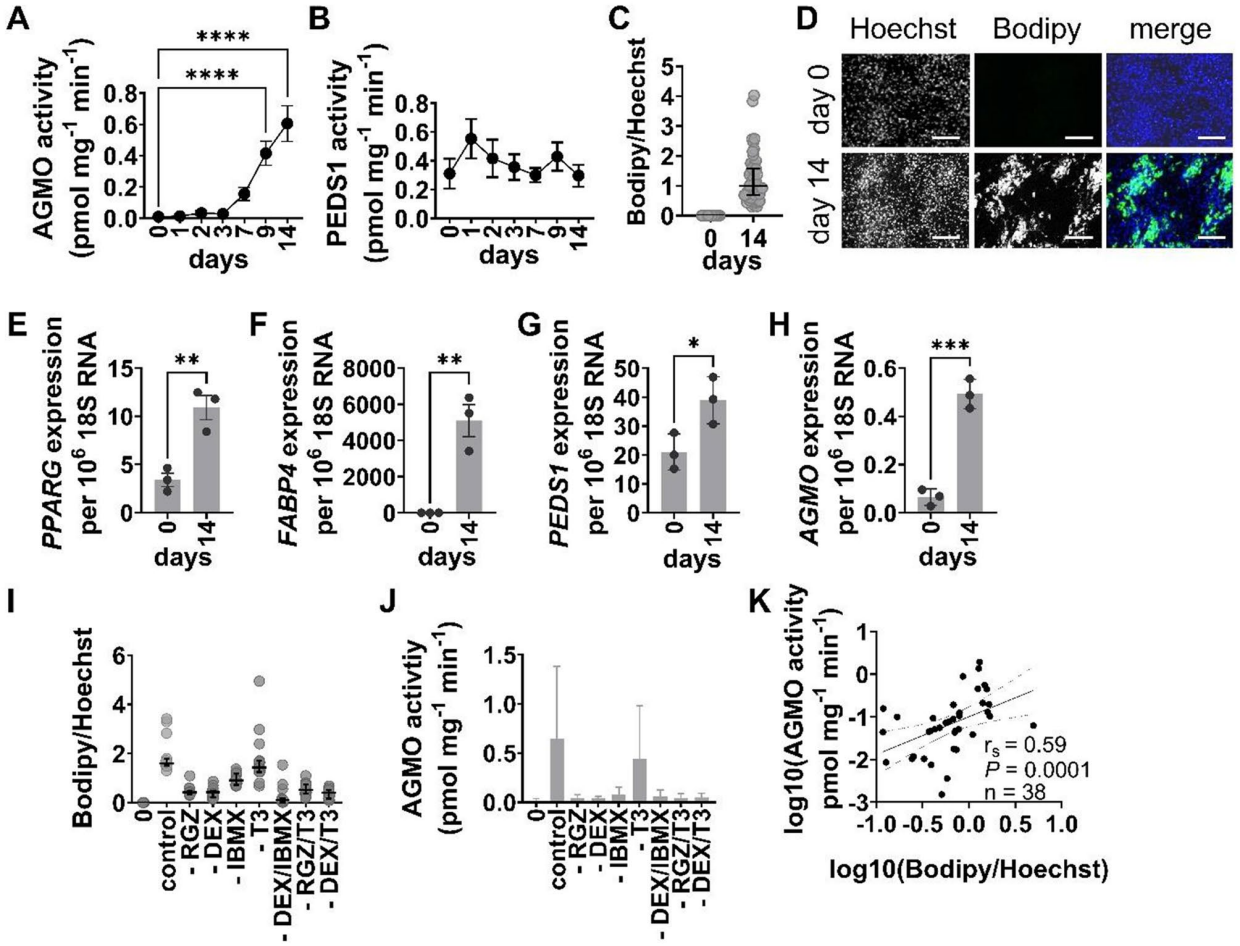
Characterization of AGMO and PEDS1 during adipose in vitro differentiation of human ASC and the influence of pro-adipogenic supplements in the induction medium. **(A)** AGMO enzyme activity was analyzed by a fluorescence-based HPLC assay [29] in cell pellets of ASC prior to exposure of the differentiation medium at day 0 and up to day 14 of in vitro adipocyte differentiation (*n* = 23). **(B)** Measurement of PEDS1 enzyme activity by a fluorescence-based HPLC assay [31] in cell pellets of ASC before exposure to differentiation at day 0 and up to day 14 of in vitro adipocyte differentiation (*n* = 12). **(C)** CellProfiler evaluation of lipid droplets (Bodipy) and nuclei (Hoechst) staining at day 0 and day 14 of adipocyte differentiation is shown. Three areas per well were recorded for each replicate. Data shows median ± IQR (*n* = 16–18). **(D)** One representative picture of Bodipy and Hoechst staining per condition. Scale bar = 250 μm. Gene expression levels of **(E) ***PPARG*, **(F) ***FABP4*, **(G) ***PEDS1* and **(H) ***AGMO* using Taqman technology are shown (*n* = 3). Data is presented as mean ± SEM. **(I)** Evaluation of Bodipy/Hoechst staining by the CellProfiler image analysis software when selected pro-adipogenic supplements were omitted (indicated by a “-” before the respective compound(s)). Three areas per well per replicate were recorded and evaluated. Data is presented as median ± IQR, *n* = 5. **(J)** AGMO enzyme activity was analyzed at day 0 and day 14 of cells exposed to the modified induction medium without selected supplements (indicated by a “-” before the respective compound(s)). Data is presented as mean ± SEM, *n* = 5. **(K)** Spearman rank correlation between AGMO activity and lipid droplet content quantified in panels **(I)** and **(J)***, n* = 38 XY pairs

We set out to determine whether AGMO and PEDS1 enzymatic activities are regulated during in vitro adipogenesis of human ASC. Therefore, we collected cell pellets on days 0, 1, 2, 3, 7, 9 and 14 during the differentiation process and quantified enzyme activities by HPLC. AGMO activity was not measurable at the beginning of differentiation, started to increase at day 7 and reached significantly elevated levels at day 9 and 14 of adipocyte differentiation compared to day 0 (Fig. 1A). PEDS1 activity was measured robustly in undifferentiated ASC and remained quite constant throughout adipocyte differentiation (Fig. 1B). Lipid droplet formation was quantified by Bodipy staining, normalized to cell number by nuclei staining with Hoechst, at day 0 and day 14 (Fig. 1C-D). Also, expression of the adipocyte markers peroxisome proliferator-activated receptor gamma (*PPARG*) and fatty acid binding protein 4 (*FABP4*) was significantly increased at day 14 (Fig. 1E-F) as well as *PEDS1* and *AGMO* gene expression (Fig. 1G-H; *PEDS1*: 2-fold, *P* = 0.005; *AGMO*: 8-fold, *P* = 0.005). No significant differences were detected between cells from male and female donors (Figure S1A and B). To assess the impact on differentiation capacity and AGMO activity, we removed specific supplements from the adipocyte induction medium. The significant decrease in lipid droplet formation after omission of all the individual factors or their combinations (rosiglitazone (RGZ), dexamethasone (DEX), DEX/3-isobutyl-1-methylxanthine (IBMX), RGZ/thyroid hormone (T3) and DEX/T3), was mirrored by a significant decline in AGMO enzyme activity (Fig. 1I-J, except for IBMX, where a higher variability in enzyme activities was present, see supplemental Table S3). Absence of T3 had no impact on enzyme activity or differentiation. We found a significant and positive correlation between AGMO activity levels and the number of lipid droplets (Fig. 1K). Concomitantly with the decrease in lipid droplet formation, there was also a 2–5 fold reduction in the expression of both *PPARG* and adiponectin (*ADIPOQ*) (Fig. S2A-C).

**Fig. 2:**
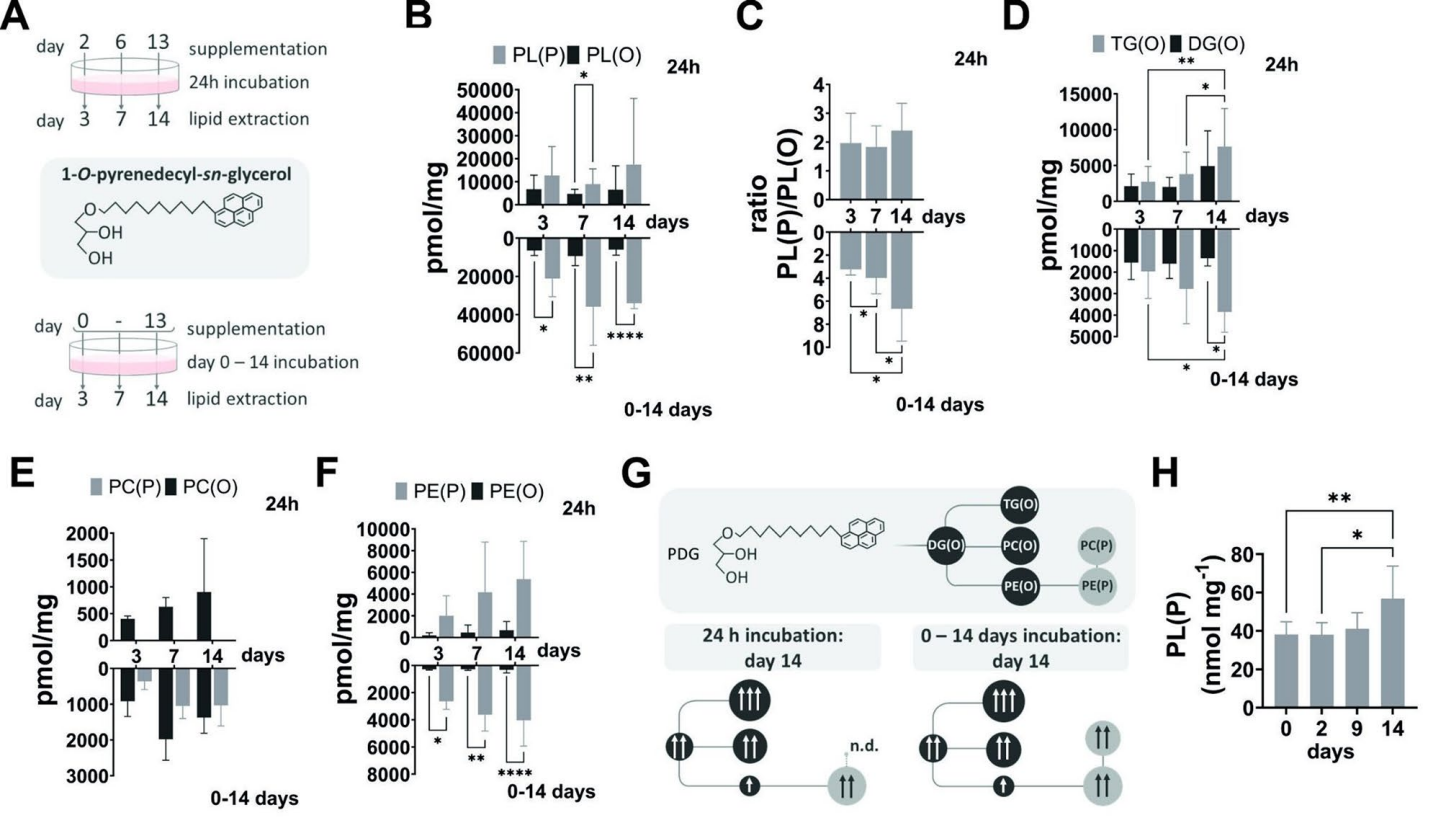
Tracking ether lipid metabolism by supplementation with the precursor 1-*O*-pyrenedecylglycerol during in vitro differentiation of human ASC. **(A)** Human ASC were supplemented with 5 µM 1-*O*-pyrenedecylglycerol for 24 h (top) or from day 0–14 (bottom) during in vitro differentiation, cell pellets were collected at indicated time points and lipids were extracted and analyzed by reversed-phase HPLC according to [31]. Bar charts show labelled **(B)** plasmanyl and plasmenyl phospholipids and **(C)** their ratio. Crude lipid extracts were further solid phase-separated and **(D)** neutral ether lipids (alkyl-diacylglycerol, TG(O) and alkyl-monoacylglycerol, DG(O)), **(E)** ether-linked phosphatidylcholine (PC(O) and PC(P)) and **(F)** ether-linked phosphatidylethanolamine (PE(O) and PE(P)) were determined by HPLC analysis. The scheme in **(G)** summarizes the results from D-F. PC(P) formation was not detected (= n.d.) during the 24 h pulsed-chase experiment. **(H)** Dansylhydrazine derivatization of aldehydes released from plasmalogens after acid treatment during the course of in vitro differentiation. Data is presented as mean ± SD, n_B−C_ = 6, n_D−F_ = 3, n_H_ = 6–10 different donors. P-values were FDR corrected

**Fig. 3:**
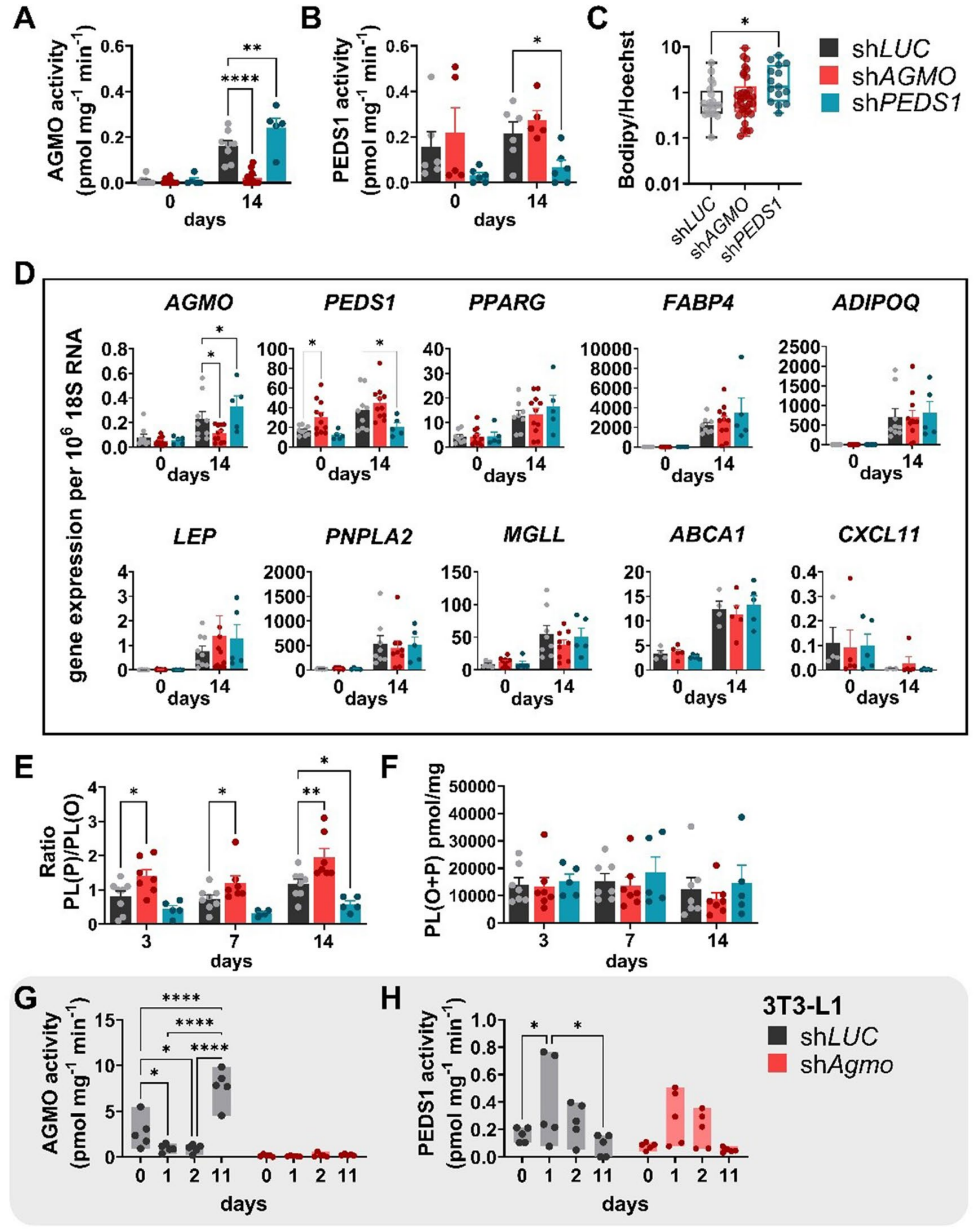
Enzyme activities and gene expression analysis of *AGMO* and *PEDS1* KD cells analyzed during in vitro adipocyte differentiation. **(A)** AGMO enzymatic activity at day 0 and day 14 during differentiation of human ASC transduced with either an shRNA against an unrelated gene as control (sh*LUC*, black bars, *n* = 8), against the *AGMO* gene (sh*AGMO*, *n* = 12 red bar), or against the *PEDS1* gene (sh*PEDS1*, blue bar, *n* = 5) is shown. **(B)** Analysis of PEDS1 enzyme activity at day 0 and day 14 during differentiation of sh*LUC* (black bars, *n* = 6), sh*AGMO* (red bars, *n* = 5) and sh*PEDS1* (blue bars, *n* = 6) cells. Data is shown as mean ± SEM. **(C)** Bodipy staining of lipid droplets and nuclear staining with Hoechst to assess lipid droplet formation (sh*LUC* open black bar: *n* = 8; sh*AGMO* red bar: *n* = 11; sh*PEDS1* blue bar: *n* = 5). Boxplots show the median ± IQR and whiskers range from minimum to maximum. **(D)** Gene expression analysis at day 0 and day 14 of *AGMO*, *PEDS*, adipocyte specific markers and adipokines such as *PPARG*,* FABP4*,* ADIPOQ* and *LEP* as well as lipolytic genes *PNPLA2 and MGLL* (sh*LUC* black bars: *n* = 8; sh*AGMO* red bars: *n* = 12; sh*PEDS1* blue bars: *n* = 5) as well as *ABCA1 and CXCL11 (*sh*LUC* black bars: *n* = 4; sh*AGMO* red bars: *n* = 4; sh*PEDS1* blue bars: *n* = 4) are shown. **(E)** 24-hour pulsed PDG feeding during differentiation. Lipids were extracted and analyzed by reversed-phase HPLC according to [31]. Bar charts show the total amount of ether-linked phospholipids (PL(O + P)) and **(F)** the ratio of plasmenyl (PL(P)) to plasmanyl (PL(O)) phospholipids during in vitro differentiation of human ASC (*n* = 5–8). Data is presented as mean ± SEM. **(G)** AGMO activity during distinct time points of 3T3-L1 shLUC and sh*Agmo* cell lines during adipocyte differentiation. Floating bars stretch from maximum to minimum (*n* = 5). **(H)** PEDS1 activity of 3T3-L1 shLUC and sh*Agmo* cell lines during adipocyte differentiation. Floating bars stretch from maximum to minimum (sh*LUC n* = 5, sh*Agmo*: sh*Agmo*506 *n* = 2 and sh*Agmo*1699 *n* = 3)

To track ether lipid formation rates during adipocyte conversion of human ASCs, we used an ether lipid precursor labelled with a fluorescent moiety (1-*O*-pyrenedecylglycerol (PDG)) and conducted a 24-hour pulse chase experiment or supplemented PDG from day 0–14 during differentiation (Fig. 2A). Our findings demonstrate that plasmenyl phospholipid (PL(P)) synthesis exceeded that of plasmanyl phospholipids (PL(O)) (Fig. 2B) reaching statistical significance at day 7 of differentiation (*P* = 0.042) during the 24-hour treatment period as compared to the 14-day feeding where PL(P) where significantly higher throughout the selected time points (day 3: *P* = 0.02; day 7: *P* = 0.006; day 14: P  < 0.0001). The ratio of PL(P) to PL(O) remained constant throughout the process of adipocyte differentiation for 24-hour supplementation whereas PDG feeding from day 0 to day 14 resulted in increased PL(P)/PL(O) ratios (day 3 vs. day 7: *P* = 0.048; day 3 vs. day 14: *P* = 0.01; day 7 vs. day 14: *P* = 0.01; Fig. 2C). Using solid phase extraction, we quantified the ether lipid content of neutral lipids, along with phosphatidylcholine (PC) and phosphatidylethanolamine (PE) glycerophospholipids. Neutral lipids showed a significant increase in alkyl-diacylglycerols (TG(O)) at the later phase of adipogenesis (3.6 ± 1.4 fold change day 14 vs. day 3, *P* = 0.004 and 2.5 ± 1.8 fold change day 14 vs. day 7, *P* = 0.026; Fig. 2D), while alkyl-monoacylglycerols (DG(O)) showed a trend to increased levels at day 14 which was not significant. PDG feeding for 14 days resulted in an unchanged amount of DG(O), whereas TG(O) levels increased comparable to the 24-hour pulse chase experiment (Fig. 2D). PC(O) was synthesized preferentially over PC(P), which were below the detection limit (Fig. 3E upper panel), while PE(P) was more abundant than PE(O) (Fig. 2F upper panel). In contrast to the 24-hour incubation, PC(P) was now also detected (Fig. 2E lower panel) while ether-linked PE was still almost exclusively present as PE(P) (Fig. 2F lower panel). The observations of ether lipid formation by PDG supplementation are summarized in Fig. 2G. Increased synthesis of PL(P) during later stages of adipocyte differentiation was also confirmed by measuring their intracellular levels using dansylhydrazine derivatization of aldehydes released from all vinyl ether bond-containing PL by acid treatment (Fig. 2H).

Furthermore, we analyzed gene expression of enzymes involved in ether lipid metabolism together with differentiation-associated genes *PPARG*, *FABP4*, *ADIPOQ* and *LEP*, which were all strongly induced (Figure S3A-D). The anabolic enzymes *GNPAT*, *AGPS*, *PEDS1* and *FAR1* were not significantly regulated during adipocyte conversion (Fig. S3E-H). In contrast, the catabolic enzymes *AGMO*, adipocyte triglyceride lipase (*PNPLA2*) and monoacylglycerol lipase (*MGLL*) were all upregulated at day 14 (Figure S3I-K). Gene expression of GTP-cyclohydrolase (*GCH1*), the rate-limiting enzyme for tetrahydrobiopterin (BH4) synthesis, the crucial co-factor for AGMO, was found to be significantly lowered at day 7 of adipogenesis, followed by a subsequent increase back to initial levels at day 14 (Fig. S3L).

### AGMO and PEDS1 knockdown alter ether phospholipid homeostasis but do not influence adipocyte differentiation

To assess the impact of *AGMO* and *PEDS1* depletion on the conversion of human ASC to adipocytes, we individually knocked down both enzymes by lentiviral transduction and exposed the manipulated cells to the adipogenic induction medium. For AGMO, we used two constructs and were able to reduce AGMO activity at day 14 by 85.85 ± 5.91% compared to control cells (*P* < 0.0001) (Fig. 3A; individual values of each knockdown construct are shown in the lentiviral transduction methods section and KD efficiency is reported in supplemental Table S4). For *PEDS1* knockdown (KD) constructs, only sh*PEDS1*940 robustly reduced the enzymatic activity by 70 ± 12%, *P* = 0.02 (Fig. 3B; and supplemental Table S4 for single values). Of note, AGMO activity in in vitro differentiated ASC with *PEDS1* KD was 1.5 fold higher as compared to the sh*LUC* control at day 14. Lipid droplet formation (Fig. 3C) was not affected by knockdown of *AGMO*, while sh*PEDS1* cells had, on average, 2-fold more lipid droplets as compared to control cells (*P =* 0.03). We also analyzed gene expression in the KD cells (Fig. 3D). Both *AGMO* and *PEDS1* were significantly reduced in the corresponding KD cells at day 14. However, gene expression of adipocyte markers and adipokines (*PPARG*, *FABP4*, *ADIPOQ*, and *LEP*) was not altered upon reduced enzyme activity. This was also true for lipolytic genes such as *PNPLA2* and *MGLL*. Additionally, *ABCA1*, a key regulator of cholesterol efflux, and *CXCL11*, an inflammation-associated chemokine, showed no significant changes in expression. To mechanistically assess the functional consequences of either reduced plasmanyl lipid catabolism by sh*AGMO* or reduced plasmalogen synthesis by sh*PEDS1*, we employed again the pulse–chase labeling approach using PDG supplementation for 24 h prior to sample collection at days 3, 7, or 14 (see Fig. 2A for the treatment scheme). Under these conditions, total ether phospholipid content did not differ significantly between groups (Fig. 3E). However, both sh*AGMO* and sh*PEDS1* cells exhibited altered PL(P)/PL(O) ratios relative to the sh*LUC* control (Fig. 3F). sh*AGMO* cells consistently displayed elevated PL(P) levels throughout differentiation, resulting in higher PL(P)/PL(O) ratios, whereas sh*PEDS1* cells showed reduced PL(P)/PL(O) ratios, reaching statistical significance at day 14 compared to the sh*LUC* control (*P* = 0.029). Further expression analysis of ether lipid relevant genes *GNPAT*, *AGPS* and *FAR1*, also showed no difference (Fig. S4A-C). *GCH1* expression was significantly higher at day 0 in the sh*AGMO* cell line (versus sh*LUC*: *P* = 0.0025 and versus sh*PEDS1*: *P* = 0.014, Fig. S4D) but declined at day 14 to values comparable to the two other cell lines. To determine whether AGMO and PEDS1 enzyme activities are similarly regulated in 3T3-L1, we measured them during distinct time points of adipocyte differentiation in sh*LUC* and sh*Agmo* cells (Fig. 3G-H). As demonstrated in earlier studies [26], AGMO activity changed dynamically during differentiation (sh*LUC*: day 0 vs. day 1, *P* = 0.03; day 0 vs. day 2, *P* = 0.02; day 0 vs. day 11, *P* < 0.0001; day 1 vs. day 11, *P* < 0.0001; day 2 vs. day 11, *P* < 0.0001). In contrast, newly generated data for PEDS1 activity showed an inverse trend, peaking around day 1 (sh*LUC*: day 0 vs. day 1, *P* = 0.04; day 1 vs. day 11, *P* = 0.01), with a similar pattern observed in both control and sh*Agmo* cells despite considerable variability.

### AGMO is active in human in vivo differentiated adipocytes

**Fig. 4:**
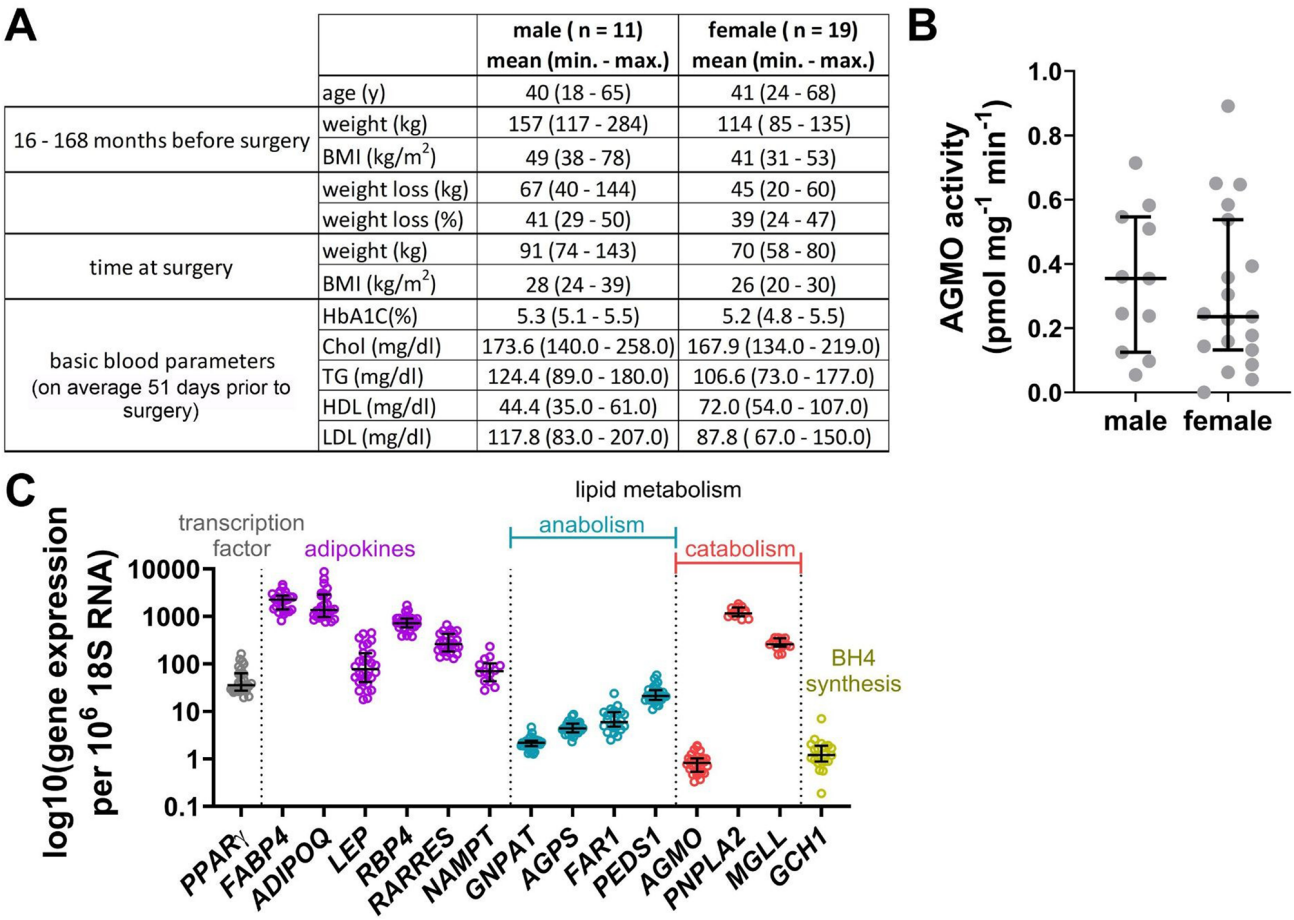
Characterization of cohort and human primary adipocyte tissue samples by AGMO activity and gene expression analysis. **(A)** Basic patient parameters including age (y), weight (kg), BMI (kg/m²) both before and at surgery, weight loss in kg or percent (*n* = 24–30) and basic blood parameters comprising haemoglobin A1C (HbA1C), cholesterol (Chol) and triglyceride (TG) levels as well as high density lipoprotein (HDL) and low density lipoprotein (LDL) concentrations (*n* = 13). Blood samples were taken on average 51 days prior to surgery (max. time = 275 days and min. = 1 day). Data is shown as mean, including minimum and maximum values in brackets. **(B)** AGMO enzymatic activity in human in vivo differentiated adipocytes was determined by HPLC [29] and separated according to gender. Data is presented as median ± IQR, male = 11 and female = 19. **(C)** Gene expression analysis of primary human adipocytes, such as *PPARG*, *FABP4*, *ADIPOQ*, *LEP*, retinol binding protein 4 (*RBP4*), retinoic acid receptor responder (*RARRES*) and nicotinamide phosphoribosyltransferase (*NAMPT/VISFATIN*), as well as lipid metabolizing genes like *GNPAT*, *AGPS*, *FAR1*, *PEDS1*, *AGMO*, *PNPLA2* and *MGLL* is shown. Additionally, expression of *GCH1*, a key gene in the AGMO cofactor tetrahydrobiopterin (BH4) biosynthesis, was analyzed as well. Data is presented as median ± IQR, *n* = 26–28

As a next step, we sought to measure AGMO and PEDS1 enzyme activities also in human whole adipose tissue samples and isolated primary adipocytes from patients who underwent abdominoplasty surgery (basic patient parameters are listed in Fig. 4A). Figure S5 illustrates the selected blood parameters from individual patients. AGMO enzyme activities were successfully measured in primary adipocytes, but not in whole tissue, while we could not detect any PEDS1 enzyme activity in primary adipocytes and whole tissue samples. There was no statistically significant difference between male and female activity data (AGMO activity median ± SEM: male 0.35 ± 0.07 pmol mg-1 min-1; female 0.24 ± 0.06 pmol mg-1 min-1; *P* = 0.58; see Fig. 4B). Gene expression analysis revealed high expression of adipocyte-related genes, including *PPARG*, and genes from the ester lipid catabolism route, such as *PNPLA2* and *MGLL* (Fig. 4C). Their median expression values were 2–3 orders of magnitude higher than those of ether lipid metabolism-related genes (*GNPAT*, *AGPS*, *FAR1*, *PEDS1* and *AGMO*).

**Fig. 5:**
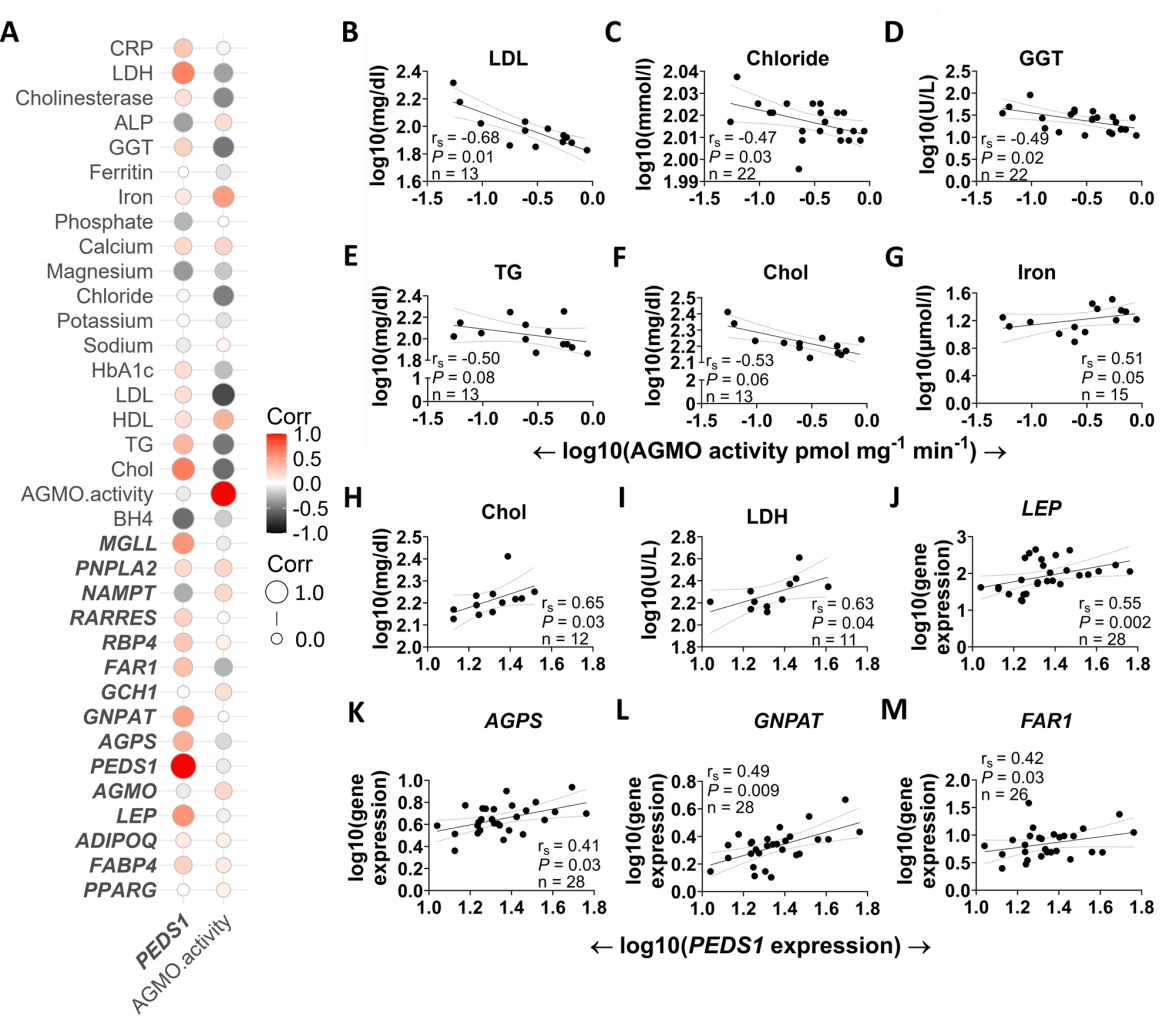
Correlation matrix of *PEDS1* gene expression and AGMO activity of human in vivo differentiated adipocytes with expression of selected genes and basic blood parameters. **(A)** A Spearman correlation matrix of *PEDS1* gene expression (left) and AGMO activity (right) with gene expression data from primary adipocytes and basic blood parameters was computed in R. The scale, which is colored from red (positive correlation) to black (negative correlation), shows the absolute value of the corresponding correlation coefficients. The size of the circles indicates the strength of each correlation. **(B) - G)** Correlation analysis of AGMO activity with basic blood parameters, including **B)** low density lipoprotein (LDL), **(C)** chloride, **(D)** gamma glutamyltransferase (GGT), **(E)** triglycerides (TG), **(F)** cholesterol (Chol) and **(G)** iron is shown. **(H - M)** Correlation analysis of *PEDS1* expression data with basic blood parameters, including **H)** cholesterol, **(I)** lactate dehydrogenase (LDH), as well as gene expression of **(J) ***LEP*, **K)*** AGPS*, **L)*** GNPAT* and **M)*** FAR1*. CRP = C-reactive protein, LDH = lactate dehydrogenase, ALP = alkaline phosphatase, HbA1c = hemoglobin A1c. For better visualization, gene names are written in bold. P-values shown are not adjusted. LFDR corrected p-values are given in Supplementary Material 2.

Correlation analysis of all measured gene expression, activity and basic blood parameters is shown in supplemental Fig. S6. For a more detailed analysis, we extracted biologically interesting correlations with r_s_ values > 0.4 that were related to basic blood parameters or to genes involved in related metabolic pathways and depict them in Fig. 5A. The full correlation matrix is available in Supplementary Material 2 and contains the correlation coefficients, p-values and local FDR-adjusted p-values.

For AGMO activity, a strong negative correlation was calculated for low-density lipoproteins (Fig. 5B), chloride (Fig. 5C) and gamma glutamyltransferase (GGT) (Fig. 5D). Furthermore, triglycerides and cholesterol were also negatively associated with AGMO activity (Fig. 5E-F). Iron was the only parameter that was positively correlated with AGMO (Fig. 5G). For *PEDS1* expression, there was a strong and positive correlation between cholesterol, lactate dehydrogenase (LDH) and *LEP* gene expression, as well as peroxisomal genes of ether lipid metabolism such as *AGPS*, *GNPAT* and *FAR1* (Fig. 5H-M).

Scatterplots for biologically meaningful correlations of *GNPAT*, *AGPS* and *FAR1* with basic blood parameters and other analyzed genes are provided in Supplemental Fig. S7-9. Gene expression of *GNPAT* was positively correlated to *FABP4*, *AGPS* and HDL. *AGPS* and *FAR1* expressions were found to strongly and positively correlate with *PPARG*, *ADIPOQ* and *RARRES*. Additionally, *AGPS* gene expression correlated positively with *FAR1*, *AGMO* and *RBP4* expression. Transferrin was negatively correlated with the expression of *AGPS* and *FAR1*. Furthermore, there was a positive correlation between *FAR1* expression and cholesterol, as well as a negative correlation with phosphate.

### Untargeted lipidomics links AGMO levels to phospholipid composition in primary in vivo differentiated adipocytes

**Fig. 6:**
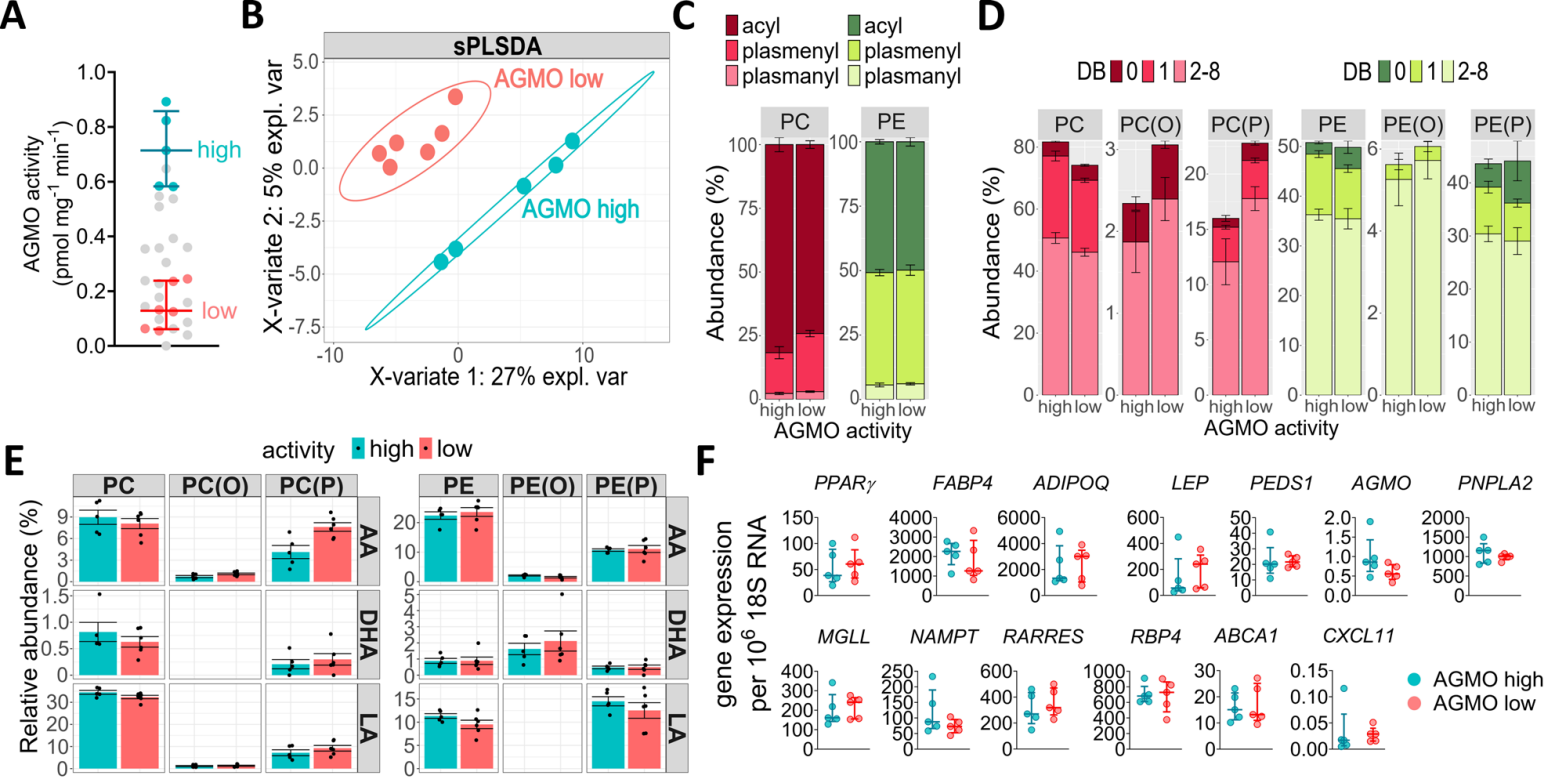
Untargeted lipidomics reveals distinct PE/PC lipid signatures in adipocytes with high vs. low AGMO activity. **(A)** Selected primary adipocytes with high (turquoise points: 0.58–0.89 pmol mg^− 1^ min^− 1^, *n* = 5) and low (coral points: 0.00-0.097 pmol mg^− 1^ min^− 1^, *n* = 6) AGMO enzyme activities as measured using the fluorescence-based HPLC activity assay (data shown in Fig. 4B). The grey points indicate AGMO enzyme activities from all other primary adipocytes that were not included in lipidomics analysis. **(B)** Sparse Partial Least-Squares Discriminant Analysis (sPLS-DA) was used for dimension reduction and separation of groups. **(C)** Percent contribution of the sum of acyl, plasmenyl and plasmanyl species in PC (magenta) and PE (green) stratified according to high versus low AGMO activity is shown. **(D)** The percent amount of double bonds (DB) found in PC (magenta) and PE (green) phospholipid subclasses is shown. Lipids were summed according to the total amount of DBs found in both *sn*-1 and *sn*-2 attached side chains. **(E)** All PC and PE species containing either LA, AA, or DHA were summed and their percent contribution to the class was calculated. LA = linoleic acid, AA = arachidonic acid, DHA = docosahexaenoic acid. For bar charts, data is presented as mean ± SEM. **(F)** Gene expression profiles of primary human adipocytes with high and low AGMO activity. Expression profiles of adipocyte-specific markers, adipokines and lipogenic as well as lipolytic genes (*PPARG*, *FABP4*, *ADIPOQ*, *LEP*, *PEDS1*, *AGMO*, *PNPLA2*, *MGLL*, *NAMPT*, *RARRES*, *RBP4*,* ABCA1 and CXCL11*). Data is presented as median ± IQR (*n* = 5). The calculated concentrations for phospholipids can be found in Supplementary Material 3

We performed lipidomics analysis only for varying AGMO activity levels and not for *PEDS1* gene expression due to the fact that, only in transgenic mice, knockout of *PEDS1* on both alleles is sufficient to affect plasmalogen levels and impact the phospholipid composition. Therefore, we selected primary adipocytes displaying low AGMO activity (range 0.00-0.097 pmol mg^− 1^ min^− 1^, *n* = 6, 2 males and 4 females) versus others that had high AGMO activity (range 0.58–0.89 pmol mg^− 1^ min^− 1^, *n* = 5, 2 males and 3 females) (Fig. 6A) and analyzed PE and PC species which are abundant in ether lipids and were regulated upon AGMO knockdown during in vitro differentiation of ASC (Fig. 3G). Using mass spectrometry-based phospholipid analysis, we were able to identify 115 single lipid species, of which 54 species were annotated to PC and 61 to PE. The application of sparse partial least-squares discriminant analysis (sPLS-DA) enabled classification of adipocytes based on their AGMO activity levels (Fig. 6B). The top 15 contributions to component 1 and component 2 are shown in supplemental Figure S10. PC(P) was significantly enriched in adipocytes with low AGMO activity levels (*P* = 0.03) that coincided with a decrease of acyl-PC (*P* = 0.05; Fig. 6C). In contrast, the composition of PE remained constant. Detailed analysis of the total double bond distribution revealed that side chains of PC(P) with 2 or more double bonds in total were increased in cells with low AGMO activity (17.8 ± 1.3% in AGMO low vs. 12.1 ± 2.1% in AGMO high; *P* = 0.046; Fig. 6D).

A detailed analysis of the side chain composition of PC and PE subclasses containing either linoleic acid (LA, 18:2), arachidonic acid (AA, 20:4) or docosahexaenoic acid (DHA, 22:6) revealed that the only change in high versus low AGMO was found for AA in PC(P) (7.6 ± 0.5% in AGMO low vs. 4.1 ± 0.8% in AGMO high; *P* = 0.009; Fig. 6E). All annotated PC species are shown in supplementary Fig. S11 and all PE species can be found in supplementary Fig. S12. At the species level, we found in total eight single species to be significantly different depending on AGMO enzyme activity levels. From these four lipid species were accumulated i.e., PC (P-16:0_14:0), PC (P-16:0_20:4), PC (P-18:0_20:4), PE (P-16:0_16:0) and four species were depleted PE (18:1_22:5), PE (18:2_16:1), PE (O-16:1_20:4), PE (O-18:1_20:1) in cells with low AGMO activity (see supplemental Table S5). We also compared gene expression between adipocytes with high and low AGMO activity (Fig. 6F). In line with our findings in late-stage in vitro–differentiated adipocytes, no substantial differences were observed in key adipocyte markers (*PPARG*, *FABP4*), adipokines (*ADIPOQ*, *LEP*), or the membrane lipid transporter *ABCA1*. *FABP4* tended to be lower in AGMO-low adipocytes, whereas *ADIPOQ* and *LEP* showed the opposite trend. Ether lipid enzymes (*PEDS1*, *AGMO*), lipolytic genes (*PNPLA2*, *MGLL*), and inflammatory adipokines and chemokines (*NAMPT*, *RARRES*, *RBP4*, *CXCL11*) likewise showed no differential expression. Mitochondrial β-oxidation, assessed via acylcarnitine profiles, also did not differ between groups (Supplementary Fig. S13). From our data, we conclude that AGMO activity has the most pronounced effect on the homeostasis of PC subclasses, specifically by changing the amount and molecular composition of PC(P).

## Discussion

Here, we investigate for the first time the role of two enzymes, AGMO and PEDS1, in human adipogenesis. Both enzymes are part of the ether lipid metabolism, a lipid pathway that has long remained understudied but has now become accessible due to recent advancements in gene annotations [14] as well as in mass spectrometric approaches [24]. Previous studies suggested that ether lipids, including plasmalogens, influence adipogenesis not only as structural components but also by regulating lipid droplet formation [38], membrane properties [39] and signaling pathways such as PPARγ activation [40, 41]. It was also shown that neutral ether lipid levels increase during adipogenesis [42], suggesting an involvement in lipid storage and metabolic regulation [43, 44]. We provide the first evidence that both enzymes are active during human ASC differentiation, showing constitutive PEDS1 activity throughout the 14-day adipogenesis protocol and induction of AGMO from day 7, independent of patient gender. This behavior of AGMO is a general feature of lipolytic genes [45, 46] and was also found by us in a former adipogenesis study performed in 3T3-L1 fibroblast cells [26]. Omission of components from the differentiation cocktail mostly led to strongly reduced adipogenesis of human ASC as judged by Bodipy staining, correlating well with decreased AGMO activity (r_S_ = 0.59, *P* = 0.0001). Our findings further underline the quality of adipocyte differentiation through the regulation of selected adipocyte-specific genes and ether lipid–specific enzymes, enabling direct comparability between the results from 3T3-L1 cells and primary human cells. Notably, reproducing our previous findings in primary cells derived from different human individuals, independent of sex, highlights the robustness of the observed AGMO regulation. However, knockdown of *AGMO* and also *PEDS1* did not interfere with differentiation itself as compared to the mock control. This was mirrored by expression of *PPARy*, *LEP* and *ADIPOQ*, indicating that both enzymes are actually dispensable for adipogenesis. This has also been demonstrated in previous studies using *Agmo* and *Peds1* KO mouse models, which present normal adipose tissue depots [17, 47]. In our current study they were, however, impacting lipid homeostasis, as both, *AGMO* and *PEDS1* knockdown, affected the PL(P)/PL(O) ratio during feeding experiments with a labelled ether lipid precursor (Fig. 3F). The data obtained on PEDS1 are, however, to be taken with caution, as the knockdown efficiency was variable across the different donor cells (on average cells still had 30% enzyme activity, see supplemental Table S4) and we know from mouse studies that mice heterozygous for a *PEDS1* knockout allele displaying 50% of wildtype activity, still present with wildtype plasmalogen levels [13, 47]. Complementary data from 3T3-L1 cells demonstrate that AGMO shows an activity pattern opposite to PEDS1, and its knockdown does not alter PEDS1 activity (Fig. 3G–H). The pronounced increase in AGMO activity in late-stage human and murine adipocytes, together with the accumulation of PC(P) following AGMO knockdown both in vitro [26] and in vivo, indicates that AGMO-dependent degradation of alkyl-ether lipids is required to maintain PL(P)/PL(O) balance. When AGMO activity is reduced, PC appears to serve as a metabolic reservoir for plasmalogen synthesis, while PE subclasses remain stable.

**Fig. 7:**
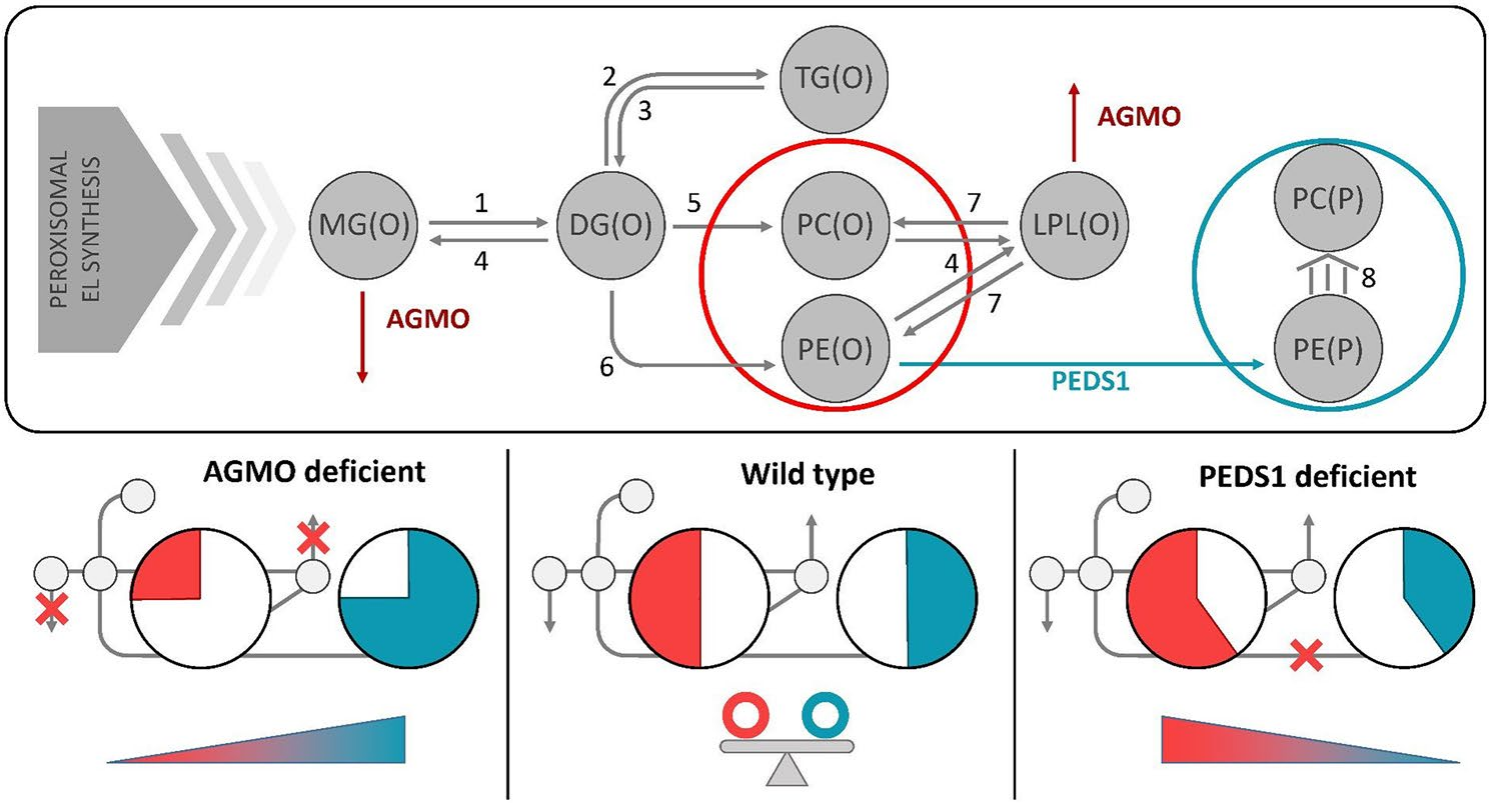
Ether lipid metabolism shaped by AGMO and PEDS1 knockdown. The upper panel shows a simplified scheme of ether lipid maturation in the endoplasmic reticulum. 1-*O*-alkyl-sn-glycerols (MG(O)) can be directly degraded by alkylglycerol monooxygenase (AGMO). Alternatively, MG(O) can serve as a substrate for monoacylglycerol acyltransferases 1 or 2 (MGAT1/2, indicated by 1) to form 1-*O*-alkyl-2-acyl-glycerols (DG(O)). DG(O) can be further converted by acyl-CoA: diacylglycerol acyltransferase 1 (DGAT1, indicated by 2) to 1-*O*-alkyl-2,3-diacyl-glycerols (TG(O)), while the reverse reaction is catalyzed by adipocyte triglyceride lipase (ATGL, indicated by 3). From DG(O), the synthesis can branch to PC(O) by choline/ethanolamine phosphotransferase 1 (CEPT1, indicated by 5) or to PE(O) by ethanolaminephosphotransferase-1 (SELENOI, indicated by 6). Plasmalogens are exclusively produced from PE(O) by plasmanylethanolamine desaturase (PEDS1), which introduces the vinyl bond in PE(P). PC(P) can be generated via three distinct pathways reviewed in detail by [15]. Plasmanyl lysophophospholipids (LPL(O)) are derived through hydrolysis of the sn-2 ester bond by phospholipase A2 (PLA2, indicated by 4), which can be further degraded again by AGMO. The reacylation of LPL(O) may be executed by lysophospholipid acyltransferases (LPLAT, indicated by 7). The lower panel highlights extraperoxisomal ether lipid profiles over 14 days of adipocyte differentiation derived from PDG pulse-chase experiments. On day 14 of differentiation, AGMO knockdown (left) leads to reduced degradation of alkyl-linked lipids, which shifts the balance from PL(O) to PL(P). Upon PEDS1 knockdown (right), total ether-linked phospholipid levels remain constant, but the reduction in PL(P) is compensated by an increase in PL(O). The middle pane shows ether lipid balance in wild-type cells. Grey routes in the upper pane denote conceptual pathways derived from literature data

Importantly, our pulse–chase experiment was designed to capture the dynamic interaction between AGMO and PEDS1 pathways in sustaining total ether lipid concentrations while fine-tuning the PL(P)/PL(O) ratios. Over longer periods, plasmalogen pools can be replenished, indicating that the transient decreases observed under reduced enzyme activity likely reflect delayed synthesis rather than a permanent loss of ether lipid content. Supplementing wildtype cells with the ether lipid precursor PDG showed that ASC also synthesized TG(O) in late-stage adipocytes in both the 24 h and 14-day feeding protocol (Fig. 2D), as previously demonstrated in various in vitro models of adipocyte differentiation, including our 3T3-L1 adipocyte lipidomics analysis [25, 26]. In general, supplementation for 14 days also increased plasmalogen levels by accumulation of PC(P) which was not seen during the 24 h feeding protocol (Fig. 2E). However, not all ether lipid subclasses were equally affected as our results revealed no significant increases in DG(O) similarly to our previous 3T3-L1 study [26]. In contrast, substantial amounts of PC(O) and PE(P) were seen (Fig. 2E-F). From data presented in Fig. 3E-F and supported by the enzymatic activity data (Fig. 3G-H) as well as lipidomics data in 3T3-L1 cells [26] the directionality of flux between PL(O) and PL(P) as well as the regulatory bottleneck created by these two enzymes is schematically outlined in Fig. 7. The role of other enzymes in this scheme has been deduced from literature.

We also looked at AGMO and PEDS1 in adipose tissue and in vivo differentiated adipocytes, but PEDS1 activity was not measurable in human adipose tissue and isolated adipocytes. Consistent with one of our earlier studies in the mouse brain, which is the organ with the highest plasmalogen content, PEDS1 activity could also not be measured with our enzyme assay [31]. Therefore, the complex matrix of adipocyte homogenates rendered this assay unsuitable for reliable activity determination in this sample type. AGMO activity, in contrast, could be detected in adipocytes but not in the tissues. Despite the challenges, we were successful in retrieving expression values for both *PEDS1* and *AGMO*. White adipose tissue distribution and function differ by sex, with females accumulating more subcutaneous (SAT) and males more visceral adipose tissue (VAT). Alongside estrogen protection, obese men exhibit lower insulin sensitivity, higher glucose levels, and reduced adiponectin compared to obese women, promoting intra-abdominal fat and insulin resistance [48]. Despite this, most in vivo obesity studies use male mice. Therefore, we also looked at potential sex differences, however, no differences between male and female samples were detected. Available data from the patients undergoing surgery allowed correlation analyses of *PEDS1* expression and AGMO activity with gene expression data and basic blood parameters and we found that *PEDS1* gene expression was positively correlated with cholesterol, leptin and lactate dehydrogenase while AGMO activity was positively associated with HDL levels and negatively correlated with LDL, triglycerides, and total cholesterol (Fig. 5). A recent study presenting a detailed single cell atlas for human adipose tissue identified *AGMO* expression in human hAd7 adipocytes - a unique subpopulation of subcutaneous adipose tissue and linked to insulin resistance, revealing a potential depot-specific role for AGMO in metabolic regulation [49]. Consistently, AGMO activity negatively correlated with GGT, a marker associated with dyslipidemia, liver injury, and metabolic syndrome [50].

We compared the lipidomic adaptation of primary human in vivo differentiated adipocytes with low vs. high AGMO activity with a focus on phospholipids (Fig. 6A). Using untargeted mass spectrometry, we could annotate 115 PC and PE species. Strong effects in cells with low AGMO activity were found in PC subclasses, manifesting in overall elevated PC(P) species with a higher number of double bonds, especially species carrying arachidonic acid as one side chain substituent (Fig. 6C-D). In contrast to mouse sWAT [47], human adipose tissue contains several ether-linked PC species, as reported in a separate study [35], highlighting species-specific differences in ether lipid composition. Plasmalogens are rich in polyunsaturated FAs, especially AA and DHA [51], and lipidomic findings from HDL reported that the major lipid constituent is PC (35–50%), including polyunsaturated PC and PC(P) species, carrying also AA (reviewed in [52]). The activity of AGMO is highest in the liver of rats and mice, but it is also active in adipose tissue [17, 29]. Therefore, AGMO in adipocytes might regulate extra-hepatocellular availability of PC(P) containing AA. In contrast, the PE subclasses remained constant regardless of AGMO activity. This tight regulation of ether PEs has already been shown in previous experiments on RCDP patient-derived fibroblasts and brain tissues from *Gnpat* KO mice [53]. Such a mechanism might be important in compensating for the loss of plasmalogens and maintaining an adequate phospholipid composition. Untargeted metabolomics analysis during leptin replacement in patients homozygous for loss-of-function mutations in *LEP* revealed a reduction in plasmalogen levels amongst other phospholipid classes [54]. Evidence is present that plasmalogen synthesis is involved in (i) facilitating cellular lipid storage and lipid droplet stability of adipose tissue [55], (ii) regulation of cholesterol biosynthesis by influencing stability of squalene monooxygenase [56] and (iii) positively influencing HDL-mediated cholesterol efflux [57] as well as uptake [58] and may be therefore linked to plasma cholesterol. Our data complements previous findings by highlighting a role for AGMO and PEDS1 in obesity and adipocyte biology, which has been only indirectly provided by plasmalogen analysis over the last years. Nonetheless, the underlying mechanisms remain largely undefined and should be addressed in future research.

## Conclusion

By analyzing samples from metabolically healthy donors (average BMI 26, HbA1c < 5.7%), our study establishes a valuable baseline for investigating AGMO and PEDS1. In light of existing evidence linking dysregulation of ether lipids to obesity [6], cardiovascular disease [59] and energy homeostasis [20] (reviewed in [38]), our findings contribute useful insights and open 

new avenues for targeted research into the function of AGMO and PEDS1 in adipocyte biology and metabolic regulation. 

Future studies should explore the following critical questions to deepen our understanding of AGMO and PEDS1: (1) Do AGMO and PEDS1 influence reverse cholesterol transport and the development of coronary artery disease? (2) How do these enzymes contribute to obesity and metabolic syndrome? (3) Is prostaglandin synthesis in sWAT specifically regulated by AGMO and PEDS1 during metabolic dysregulation?

Addressing these questions will require the implementation of relevant in vivo models, including ApoE- or LDL receptor–deficient mice and diet-induced hypercholesterolemia models to study lipid transport and atherosclerosis. For metabolic phenotyping, high-fat or Western diet feeding, as well as leptin-deficient (ob/ob) or db/db mouse models, will enable systematic investigation of obesity and metabolic syndrome. 

## Supplementary Information

Below is the link to the electronic supplementary material.

Supplementary Material 1

Supplementary Material 2

Supplementary Material 3

## Data Availability

All data generated or analyzed during this study are included in this published article (Supplemental Material and Methods, Supplemental Figures, Supplemental Tables and Additional Excel files).

## Abbreviations

ASC: Adipose-derived stem cells
*ADIPOQ*: Adiponectin
*AGMO*: Alkylglycerol monooxygenase
*AGPS*: Alkylglycerone phosphate synthase
DG(O): Alkyl-acylglycerol
*FABP4*: Fatty acid binding protein 4
*FAR1/2*: Fatty acyl-CoA reductase 1/2
GGT: Gamma glutamyltransferase
*GCH1*: GTP cyclohydrolase 1
*GNPAT*: Glycerone phosphate acyltransferase
HDL: High density lipoprotein
KD: Knockdown
LDH: Lactate dehydrogenase
LDL: Low density lipoprotein
*LEP*: Leptin
*MGLL*: Monoacylglycerol lipase
*NAMPT/VISFATIN*: Nicotinamide phosphoribosyltransferase
*PEDS1*: Plasmanylethanolamine desaturase
PE(O): Plasmanyl-phosphatidylethanolamine
PE(P): Plasmenyl-phosphatidylethanolamine
PC(O): Plasmanyl-phosphatidylcholine
PC(P): Plasmenyl-phosphatidylcholine
PL: Phospholipid(s)
*PNPLA2*: Adipocyte triglyceride lipase
*PPARG*: Peroxisome proliferator activated receptor gamma
*RARRES*: Retinoic acid receptor responder
*RBP4*: Retinol binding protein 4
TG(O): Alkyl-diacylglycerol
sPLS-DA: Sparse Partial Least-Squares Discriminant Analysis
