# Supplementary material for: Make or break - PEDS1 and AGMO orchestrate ether lipid homeostasis in human adipocytes and are associated with blood lipid profiles
Source: J Transl Med. 2026 Jan 24;24:262. doi: 10.1186/s12967-026-07728-8 (PMC12910994; doi:10.1186/s12967-026-07728-8)
Supplement: Supplementary file 1 — Supplementary Material 1 [file 12967_2026_7728_MOESM1_ESM.docx]

## Supplemental Information

## Supplemental Material and Methods

### Isolation of human adipose tissue-derived stem cells and *in vivo* differentiated adipocytes

Subcutaneous fat tissue was retrieved from patients of elective abdominoplasty surgery, which was approved by the Ethics Committee of the Medical University of Innsbruck (AN2014-0244 341/4.5 388/5.5). Written informed consent was obtained from all donors and the methods were carried out in accordance with the approved guidelines. Human adipose stem cells (ASC) and *in vivo* differentiated adipocytes were isolated according to Morandi et al. [[1]](https://paperpile.com/c/hQgD64/Sc2t).

Adipose tissue was washed with phosphate-buffered saline (1x PBS), minced into pieces, and incubated with collagenase type 2 (0.15% in PBS, Roche, Mannheim, Germany) at a ratio of 1:3 (w/v) for 1 h at 37 °C. Homogenates (150 - 200 ml) were centrifuged at 500 × g for 5 min at RT. The pelleted stromal vascular fraction (SVF) was washed twice with 1x PBS and centrifuged again at 500 x g for 5 minutes at RT. The supernatant was aspirated and the SVF pellet was incubated in erythrocyte lysis buffer (Roth, Karlsruhe, Germany) and treated for 20 min in the dark at room temperature. Thereafter, the suspension was filtered through a 100 µm nylon mesh cell strainer (VWR) and spun at 500 × g for 5 min at RT. The SVF pellet was resuspended in DMEM/F12 medium (PAN-Biotech, Aidenbach, Germany), counted with a CASY^TM^ cell counter (Schärfe System, Reutlingen, Germany) and plated at a density of 260,000 cells/cm^2^ for culture in PM4 medium [[2](https://paperpile.com/c/hQgD64/PUQc)] containing DMEM/F12 (PAN Biotech) supplemented with 1 ng/ml recombinant human fibroblast growth factor 2 (rhFGF2) (Immunotools, Frisoythe, Germany), 10 ng/ml epidermal growth factor (EGF) (Immunotools), 50 ng/ml insulin (Roche), 2.5% FCS (Fisher Scientific, Vienna, Austria) and 1% penicillin/streptomycin (GE Healthcare, Freiburg Germany). One hour after plating, non-adherent cells were washed off. 50 ml of the homogenate was used for the isolation of *in vivo* differentiated adipocytes and filtered through a 200 µm nylon mesh cell strainer (VWR, Vienna, Austria). *In vivo* differentiated adipocytes were phase separated by gravity and washed with 1x PBS until the lower phase was clear. Afterwards, adipocytes were aliquoted, snap frozen in liquid nitrogen and stored at -80°C until analysis.

### Adipocyte differentiation of human ASC and staining of lipid droplets

After isolation, adipose tissue-derived stem cells were plated at an initial density of 6 x 10^5^ cells / well of a 6-well plate or 1.2 x 10^5^ cells / well of a 24-well plate and the following day, adipocyte differentiation was induced by adding an adipogenic induction medium according to [[1]](https://paperpile.com/c/hQgD64/Sc2t) with some minor modifications. For differentiation DMEM/F12 medium supplemented with 2.5% FCS, 1 μM rosiglitazone (RGZ, Cayman, Tallinn, Estonia), 500 μM 3-isobutyl-1-methyl-xanthine (IBMX, Sigma, Vienna, Austria), 250 nM triiodothyronine (T3, Sigma), 100 nM dexamethasone (DEX, Sigma) and 1 μM insulin (Sigma). After 7 days, the medium was exchanged for adipocyte differentiation medium containing 2.5% FCS, 1 μM insulin and 1 μM rosiglitazone and cells were cultured for a further 7 days until day 14. Lipid droplets of *in vitro* differentiated adipocytes were stained with Bodipy™ 493/503 (Fisher Scientific) and nuclei were stained with Hoechst (H 33342, Merck, Darmstadt, Germany).

For staining and quantification of lipid droplets, cells were washed once with 1x PBS and fixed for 10 minutes in 4% paraformaldehyde (Merck). After fixation, cells were washed twice with 1x PBS and then incubated for 15 min in the dark at RT with a staining solution consisting of 2 µM Bodipy, 2 µg/ml Hoechst in 1x PBS. Thereafter, cells were again washed twice and then stored in 1x PBS at 4°C in the dark until image acquisition on a Leica M205 FA stereo fluorescence microscope (Leica, Wetzlar, Germany). All images were evaluated using the CellProfiler™ (2.1.1) cell image analysis software. For Bodipy/Hoechst evaluation, integrated intensities of the dyes were multiplied by their respective counts and afterwards the ratio was calculated. Undifferentiated cells were seeded at 50 % confluency and kept in growth medium (PM4 medium) in parallel to differentiating cells.

### RNA isolation and gene expression analysis

Total RNA from undifferentiated and differentiated ASC cells was prepared using the Monarch Total RNA Miniprep kit according to the manufacturer’s protocol (NEB, Frankfurt, Germany). Total RNA from *in vivo* differentiated human adipocytes was isolated using the RNeasy Lipid Tissue Mini kit (Qiagen, Hilden, Germany) according to the manufacturer’s protocol with a minor modification (see Supplemental Material and Methods). After homogenization of primary adipocytes with the QIAzol^TM^ Lysis Reagent, samples were centrifuged at 12,000 g for 10 minutes at 4°C and the upper fatty layer was aspirated prior to the addition of chloroform. On-column DNA digestion was done for all samples using the DNase included in the Monarch Total RNA Miniprep kit and the RNeasy Lipid Tissue Mini kit. Transcription into cDNA was performed for all RNAs using the M-MLV reverse transcriptase (RNase H Minus, Point Mutant; Promega, Mannheim, Germany) and random hexamer primers (Microsynth, Balgach, Switzerland). For qPCR, the TaqMan assay technology using Brilliant III Ultra-Fast QPCR Master Mix (Agilent Technologies, Vienna, Austria) and the Mx3005P qPCR system (Agilent) were used. Taqman probes were labelled with FAM (5’) and TAMRA (3’). Primer and Taqman probe sequences are listed in Supplemental Table S1.

### Enzyme activity assays

AGMO enzyme activity was measured as described in a previous work [[3]](https://paperpile.com/c/hQgD64/hjV0) with the following modifications: Homogenates of differentiated and undifferentiated ASC were not centrifuged and a protein concentration of ≥ 1 mg/ml was used to measure the enzymatic activity. Furthermore, fatty aldehyde dehydrogenase, essential for full conversion of the fatty aldehyde to the fatty acid, was added in its recombinant form to the assay mixture [[4]](https://paperpile.com/c/hQgD64/0bOyN). PEDS1 enzyme activity was measured as described in a previous work [[5]](https://paperpile.com/c/hQgD64/xdah).

For the determination of AGMO enzymatic activity in primary *in vivo* differentiated adipocytes a protease inhibitor cocktail was added, consisting of: 1 µg/ml aprotinin, 2 mM iodoacetamide, 1 µg/ml leupeptin, 50 µM benzamidine and 1 µM pepstatin A (all purchased from Sigma). One ml of frozen sample was quickly thawed, homogenized using a high-speed stirrer (ULTRA-TURRAX® IKA, Staufen, Germany) and centrifuged at 10,000 g for 1 minute at room temperature. The lower phase was used for further analysis. We carefully analyzed samples and controls of each replicate in parallel to exclude artefacts due to the day-to-day variability of the assay. PEDS1 enzyme activity was measured with the following minor modifications: Homogenates of differentiated and undifferentiated ASC were not centrifuged, and a protein concentration of 1 mg/ml was used to measure the enzymatic activity.

### Lentiviral transduction of human ASC

Generation of pHR-SFFV-PURO plasmid was described previously [[6]](https://paperpile.com/c/hQgD64/2rCW) and cloning of short hairpin (sh) RNAs into the lentiviral transduction plasmid pHR-DEST-SFFV-Puro was performed according to [[7]](https://paperpile.com/c/hQgD64/w4PH) and contained the shRNA-encoding oligonucleotides of either human AGMO ((i) 343-361 for sh*AGMO*343 or (ii) 1699-1717 for sh*AGMO*1699 (GeneBank accession no. NM_001004320.2)) or the human *PEDS1* gene ((iii) 928-946 for sh*PEDS1*928 or (iv) 940-958 for sh*PEDS1*940 (GeneBank accession no. NM_199129.3)). For the generation of lentiviral particles, 1.5 μg of sequence-verified plasmids was co-transfected with 0.9 μg pSPAX2 packaging and 0.9 μg pMD-G (VSV-G-) pseudotyping plasmids using calcium phosphate transfection. Supernatants were harvested 48 h and 72 h after transfection, filtered (pore size < 0.2μm), and diluted 1:2 with fresh PM4 medium supplemented with 1 μg/ml polybrene for infection. Puromycin (1 μg/ml) was added 48 h after infection for selection. All reagents were obtained from Sigma Aldrich. The sh*LUC* control cell line expressing shRNA against luciferase (155-173 from pGL3 Luciferase; Promega) was generated in parallel. For sh*AGMO*343, there was an 80.32 ± 7.99% AGMO activity reduction (P < 0.0001) and a 95.54 ± 3.97% AGMO activity reduction (P < 0.0001) for sh*AGMO*1699. For sh*PEDS1*928, the enzymatic activity was even higher in two out of three replicates compared to the control cell line and was therefore excluded (sh*PEDS1*928: 17 ± 51% PEDS1 activity reduction).

### Mass spectrometric analysis of phospholipids and acylcarnitines

For phospholipid analysis, an Agilent InfinityLab Poroshell 2.7 µm (120 EC-C8; 2.1x100 mm) (Agilent) was used. A Vanquish UHPLC system (Thermo Fisher Scientific, Waltham, MA, USA) was coupled to a time of flight Pro ion mobility mass spectrometer equipped with a VIP-HESI source (Bruker, Bremen, Germany). The mobile phase consisted of (A) 60:40 (v:v) acetonitrile:water and (B) 90:10 (v/v) isopropanol:acetonitrile, each containing 0.2 % formic acid and 10 mM ammonium formate. The flow rate was set to 0.4 ml/min, the LC gradient consisted of: isocratic at 40% B at 0 min, ramp to 65% B at 20 min, ramp to 99% B at 22 min, ramp to 40% B at 26 min and ramp to and isocratic at 40% B for 26 – 28 min. The flow was increased to 0.5 ml/min at 22 – 25 min. A detailed description of mass spectrometry parameters can be found in Supplemental Table S2. In brief data data-dependent acquisition- parallel accumulation-serial fragmentation (DDA-PASEF) was used and operated in negative ion mode. Untargeted phospholipid analysis was done with Metaboscape2021b (Bruker). To confirm plasmalogen annotations, samples were compared with lipid extracts from *Peds1* knockout mice that were measured in parallel. Annotated lipids were afterwards verified by linear regression analysis of published retention times using the annotation list from Lange et al. [[8]](https://paperpile.com/c/hQgD64/38hi). To confirm plasmalogen annotations, samples were compared with lipid extracts from *Peds1* knockout mice that were measured in parallel. For acylcarnitine analysis the column temperature was set to 30°C. The mobile phase consisted of (A) 100% water and (B) 90:10 (v/v) acetonitrile:water, each containing 0.1 % formic acid (VWR) and 5 mM ammonium formate (VWR). The flow rate was set to 0.4 ml/min, the LC gradient consisted of: isocratic at 98% B at 0 min, ramp to 93% B at 0.5 min, ramp to 60% B at 7 min, ramp to 40% B at 9 min and ramp to 98% 10.5 min and isocratic at 98% B for 11.5 – 12 min. The flow was increased to 0.5 ml/min at 9.5 – 10.5 min.

## References

1. [Morandi EM, Verstappen R, Zwierzina ME, Geley S, Pierer G, Ploner C. ITGAV and ITGA5 diversely regulate proliferation and adipogenic differentiation of human adipose derived stem cells. Sci Rep. 2016;6: 28889. doi:](http://paperpile.com/b/hQgD64/Sc2t)[10.1038/srep28889](http://dx.doi.org/10.1038/srep28889)

2. [Skurk T, Ecklebe S, Hauner H. A novel technique to propagate primary human preadipocytes without loss of differentiation capacity. Obesity . 2007;15: 2925–2931. doi:](http://paperpile.com/b/hQgD64/PUQc)[10.1038/oby.2007.349](http://dx.doi.org/10.1038/oby.2007.349)

3. [Werner ER, Hermetter A, Prast H, Golderer G, Werner-Felmayer G. Widespread occurrence of glyceryl ether monooxygenase activity in rat tissues detected by a novel assay. J Lipid Res. 2007;48: 1422–1427. doi:](http://paperpile.com/b/hQgD64/hjV0)[10.1194/jlr.D600042-JLR200](http://dx.doi.org/10.1194/jlr.D600042-JLR200)

4. [Keller MA, Watschinger K, Golderer G, Maglione M, Sarg B, Lindner HH, et al. Monitoring of fatty aldehyde dehydrogenase by formation of pyrenedecanoic acid from pyrenedecanal. J Lipid Res. 2010;51: 1554–1559. doi:](http://paperpile.com/b/hQgD64/0bOyN)[10.1194/jlr.D002220](http://dx.doi.org/10.1194/jlr.D002220)

5. [Werner ER, Keller MA, Sailer S, Seppi D, Golderer G, Werner-Felmayer G, et al. A novel assay for the introduction of the vinyl ether double bond into plasmalogens using pyrene-labeled substrates. J Lipid Res. 2018;59: 901–909. doi:](http://paperpile.com/b/hQgD64/xdah)[10.1194/jlr.D080283](http://dx.doi.org/10.1194/jlr.D080283)

6. [Ploner C, Rainer J, Niederegger H, Eduardoff M, Villunger A, Geley S, et al. The BCL2 rheostat in glucocorticoid-induced apoptosis of acute lymphoblastic leukemia. Leukemia. 2008;22: 370–377. doi:](http://paperpile.com/b/hQgD64/2rCW)[10.1038/sj.leu.2405039](http://dx.doi.org/10.1038/sj.leu.2405039)

7. [Sigl R, Ploner C, Shivalingaiah G, Kofler R, Geley S. Development of a multipurpose GATEWAY-based lentiviral tetracycline-regulated conditional RNAi system (GLTR). PLoS One. 2014;9: e97764. doi:](http://paperpile.com/b/hQgD64/w4PH)[10.1371/journal.pone.0097764](http://dx.doi.org/10.1371/journal.pone.0097764)

8. [Lange M, Angelidou G, Ni Z, Criscuolo A, Schiller J, Blüher M, et al. : A reference lipidome for human white adipose tissue. Cell Rep Med. 2021;2: 100407. doi:](http://paperpile.com/b/hQgD64/38hi)[10.1016/j.xcrm.2021.100407](http://dx.doi.org/10.1016/j.xcrm.2021.100407)

## Supplemental Tables

**Table S1 TaqMan primer and probe list.** Probes were FAM (5’) and TAMRA (3’) labelled.

| **Gene** | **Forward primer** | **Reverse primer** | **TaqMan probe** |
| --- | --- | --- | --- |
| ***18S*** | 5’-CCATTCGAACGTCTGCCCTAT-3’ | 5’-TCACCCGTGGTCACCATG-3’ | 5’-ACTTTCGATGGTAGTCGCCGTGCCT-3’ |
| ***ADIPOQ*** | 5’-TCCTAAGGGAGACATCGGTGAA-3’ | 5’-CATAGGCACCTTCTCCAGGTTCT-3’ | 5’-AGGCTTTCCGGGAATCCAAGGCAG-3’ |
| ***AGMO*** | 5’-CTGACCTTGACTTCCATTGGATT-3’ | 5’-CAAGCAACGGAGAGTTTCCATA-3’ | 5’-CTTCTGGATCAAAGACCCAAGGCAGCT-3’ |
| ***AGPS*** | 5’-CGTATGTCAACAGGCCCTGAT-3’ | 5’-TTGTAGCTTCTGTTATTACACCAAGAGTT-3’ | 5’-CCATCACTTCATCATGGGATCTGA-3’ |
| ***FABP4*** | 5’-AAAGAAGTAGGAGTGGGCTTTGC-3’ | 5’-CCCCATTCACACTGATGATCAT-3’ | 5’-TTAGGTTTGGCCATGCCAGCCACT-3’ |
| ***FAR1*** | 5’-GGCAATTGTAAGGCCATCGA-3’ | 5’-TTAAAGTTATCAATCCATCCTGGAAA-3’ | 5’-TGTTGGTGCCAGTTGGAAAGAAC-3’ |
| ***GCH1*** | 5’-CATTAGGAGCTGAGCTTCATTCAG-3’ | 5’-CAGACAGACAATGCTACTGGCAGT-3’ | 5’-CGATCGGCAACCAACGCACACA-3’ |
| ***GNPAT*** | 5’-GCCCATCCTTAGTAGCAGTAGCATT-3’ | 5’-GAAGCGAAAGCAACTGTAGACATC-3’ | 5’-CAGATGACACCAGGGTTCAGGAAAGA-3’ |
| ***LEP*** | 5’-TCACCAGGATCAATGACATTTCAC-3’ | 5’-CCCAGGAATGAAGTCCAAACC-3’ | 5’-CACGCAGTCAGTCTCCTCCAAACAGAAA-3’ |
| ***MGLL*** | 5’-TGCCAATCCTGAATCTGCAA-3’ | 5’-GCAGCACAAGGTTGAGCACTT-3’ | 5’-AACTTTCAAGGTCCTTGCTGCG-3’ |
| ***NAMPT*** | 5’-TGGGCATCTTCCAATAGAAATAAAA-3’ | 5’-CCACCGTGAAGAGAACATTTCC-3’ | 5’-CTGTTCCTGAGGGCTTTGTCAT-3’ |
| ***PEDS1*** | 5’-CCCACGAGACCTACTTCTGCAT-3’ | 5’-CGTCGCCAGAAGCCTATCTT-3’ | 5’-CACAGGCTGGCTCAACTACCCTCTGG-3’ |
| ***PNPLA2*** | 5’-GAGCTCATCCAGGCCAATGT-3’ | 5’-GGTTGTCTGAAATGCCACCAT-3’ | 5’-CAGCGGTTTCATCCCCGTGTACTGTG-3’ |
| ***PPARG*** | 5’-GAAACTTCAAGAGTACCAAAGTGCAA-3’ | 5’-AGGCTTATTGTAGAGCTGAGTCTTCTC-3’ | 5’-CAAAGTGGAGCCTGCATCTCCACCTTATT-3’ |
| ***RARRES*** | 5’-TGGGAGGAAACGGAAATGC-3’ | 5’-ACCGGCCCAGAACTTTGTC-3’ | 5’-TGGCCTGCATCAAACTGGGCTCTG-3’ |
| ***RBP4*** | 5’-ACGAGACCGGCCAGATGA-3’ | 5’-CACACGTCCCAGTTATTCAAAAGA-3’ | 5’-CGCCACAGCCAAGGGCCG-3’ |
| ***ABCA1*** | 5'-GGGAGGCTCCCGGAGTT-3' | 5'-GTATAAAAGAAGCCTCCGAGCATC-3' | 5'-TGTCCACGTGTTGAGATCATTGCTACAATG-3' |
| ***CXCL11*** | 5'-CCTTGGCTGTGATATTGTGTGC-3' | 5'-CCTATGCAAAGACAGCGTCCT -3' | 5'-CAGTTGTTCAAGGCTTCCCCATGTTCA-3' |

**Table S2 Method parameters of the timsTof Pro instrument for phospholipid analysis**

| **MS component** | **Setting** |
| --- | --- |
| Source | VIP-HESI |
| Scan Mode | MS/MS (PASEF) |
| Capillary temperature | 420 °C |
| Dry heater | 260°C |
| Dry gas flow | 10 L/min |
| Capillary voltage | 4500 V |
| End plate offset | - 500 V |
| Nebulizer | 4.00 Bar |
| Sheath gas flow | 5.0 L/min |
| Full mass range | 100 m/z - 1350 m/z |
| Inverse ion mobility (1/K_0_) (TIMS) | 0.55 - 1.57 Vs/cm² |
| IMS ramp time (TIMS) | 100 ms |
| IMS imeX spectra Rate (TIMS) | 9.43 Hz |
| Ion energy (quadrupole) | - 5.0 eV |
| Low Mass (quadrupole) | 150 m/z |
| Transfer time (collision cell) | 65 µs |
| Pre pulse storage (collision cell) | 5.0 µs |
| Collision energy (collision cell) | - 10 eV |
| Collision cell RF (collision cell) | 1100.0 Vpp |
| Deflector plate delta 1 (transfer parameters) | 0.0 V |
| Deflector plate delta 3 (transfer parameters) | - 80.0 V |
| Funnel 1 and 2 RF (transfer parameters) | 250.0 Vpp |
| isCID energy (transfer parameters) | 0.0 eV |
| Multipole RF (transfer parameters) | 200 Vpp |

**Table S3 Bodipy and Hoechst staining and AGMO activity values after omission of pro-adipogenic supplements in the adipocyte induction medium.**

|  | **Bodipy/Hoechst** | | | **AGMO activity (pmol mg^-1^ min^-1^)** | | |
| --- | --- | --- | --- | --- | --- | --- |
|  | **Median** | **IQR** | **p-value** | **Mean** | **SEM** | **p-value** |
| **0** | 0 | 0 | <0.0001 | 0.015 | 0.009 | 0.0003 |
| **ctrl** | 1.579 | 0.135 | - | 0.651 | 0.293 | - |
| **- RGZ** | 0.382 | 0.067 | 0.0001 | 0.039 | 0.015 | 0.005 |
| **- DEX** | 0.533 | 0.263 | <0.0001 | 0.037 | 0.009 | 0.009 |
| **- IBMX** | 0.747 | 0.093 | 0.494 | 0.083 | 0.028 | 0.08 |
| **- T3** | 1.548 | 0.905 | >0.9999 | 0.439 | 0.216 | 0.66 |
| **- DEX/IBMX** | 0.119 | 0.019 | <0.0001 | 0.062 | 0.026 | 0.013 |
| **- RGZ/T3** | 0.493 | 0.533 | 0.001 | 0.040 | 0.019 | 0.006 |
| **- DEX/T3** | 0.537 | 0.163 | <0.0001 | 0.048 | 0.017 | 0.009 |

**Table S4 Efficiency of individual knockdowns for sh*AGMO* and sh*PEDS1* compared to the corresponding control sh*LUC* per donor calculated from enzyme activity data.**

| **Donor #** | **sh*AGMO***  **knockdown efficiency (%)** | **sh*PEDS1***  **knockdown efficiency (%)** | |
| --- | --- | --- | --- |
|  | **day 14** | **day 0** | **day 14** |
| **1** | **99.2** | **100** | **82.7** |
| **2** | **75** | **61.5** | **86.3** |
| **3** | **81.3** | **62.7** | **30.1** |
| **4** | **100** | **62.6** | **43.3** |
| **5** | **100** | **36.4** | **100** |
| **6** | **47.1** |  | |
| **7** | **61.1** |  | |
| **8** | **96.5** |  | |
| **9** | **98.6** |  | |
| **10** | **100** |  | |
| **11** | **88.4** |  | |
| **12** | **100** |  | |

**AGMO activity could only be reliably measured at day 14 of differentiation in late-stage adipocytes, whereas PEDS1 activity was detected throughout the differentiation process.**

**Table S5 Top 9 lipid species that were differentially abundant depending on high or low AGMO enzyme activities in primary *in vivo* differentiated human adipocytes.**

| **molecular species** | **P value** | **method** | **alternative (Hypothesis test)** |
| --- | --- | --- | --- |
| **PC P-16:0_14:0** | 0.01732 | Wilcoxon rank sum exact test | two.sided |
| **PC P-16:0_20:4** | 0.01732 | Wilcoxon rank sum exact test | two.sided |
| **PC P-18:0_20:4** | 0.01732 | Wilcoxon rank sum exact test | two.sided |
| **PE 18:1_22:5** | 0.04975 | Wilcoxon rank sum test with continuity correction | two.sided |
| **PE 18:2_16:1** | 0.04975 | Wilcoxon rank sum test with continuity correction | two.sided |
| **PE O-16:1_20:4** | 0.03409 | Wilcoxon rank sum test with continuity correction | two.sided |
| **PE O-18:1_20:1** | 0.01502 | Wilcoxon rank sum test with continuity correction | two.sided |
| **PE P-16:0_16:0** | 0.00866 | Wilcoxon rank sum exact test | two.sided |

**Table S6 QPCR data of in vivo differentiated adipocytes with high and low AGMO activity**

| **Gene** | **AGMO high** | | | | | **AGMO low** | | | | |
| --- | --- | --- | --- | --- | --- | --- | --- | --- | --- | --- |
| *PPARG* | 19.4 | 38.6 | 33.85 | 77.6 | 100.2 | 27.0 | 40.4 | 60.6 | 111.8 | 63.9 |
| *FABP4* | 2575.7 | 2265.9 | 2775.5 | 1111.5 | 2056.4 | 2331.1 | 3330.3 | 811.4 | 1258.1 | 1266 |
| *ADIPOQ* | 1071.8 | 1331.2 | 1218.7 | 2732.4 | 4936.3 | 774.1 | 1309.3 | 3011.6 | 3909.3 | 3069.7 |
| *LEP* | 114.2 | 26.5 | 56.97 | 449.1 | 41.5 | 63.5 | 51.1 | 241.3 | 354.9 | 265.7 |
| *PEDS1* | 40.8 | 17.7 | 20.7 | 20.2 | 10.97 | 24.5 | 26.6 | 21.6 | 18.8 | 17.95 |
| *AGMO* | 0.96 | 0.47 | 1.90 | 0.86 | 0.77 | 0.50 | 0.56 | 0.85 | 0.74 | 0.33 |
| *PNPLA2* | 799.6 | 1162.7 | 860.8 | 1498.8 | 1156.01 | 1075.1 | 1007.5 | 1009.6 | 849.99 | 993.2 |
| *MGLL* | 218.5 | 155.9 | 128.3 | 341.5 | 161.1 | 152.8 | 275.50 | 253.6 | 240.7 | 157.1 |
| *NAMPT* | 58.6 | 146.4 | 73.6 | 87.8 | 233.3 | 86.5 | 103.7 | 72.7 | 61.4 | 43.8 |
| *RARRES* | 244.8 | 147.1 | 270.3 | 460.97 | 408.9 | 228.5 | 318.6 | 440.5 | 500.01 | 297.1 |
| *RBP4* | 681.8 | 699.8 | 609.2 | 910.7 | 611.7 | 579.1 | 729.8 | 902.97 | 823.6 | 377.7 |
| *ABCA1* | 10.2 | 23.1 | 15.1 | 12.2 | 19.8 | 579.1 | 729.8 | 902.97 | 823.6 | 377.7 |
| *CXCL11* | 0.018 | 0.116 | 0.008 | 0.017 | 0.005 | 0.016 | 0.015 | 0.051 | 0.029 | 0.029 |

## Supplemental Figures


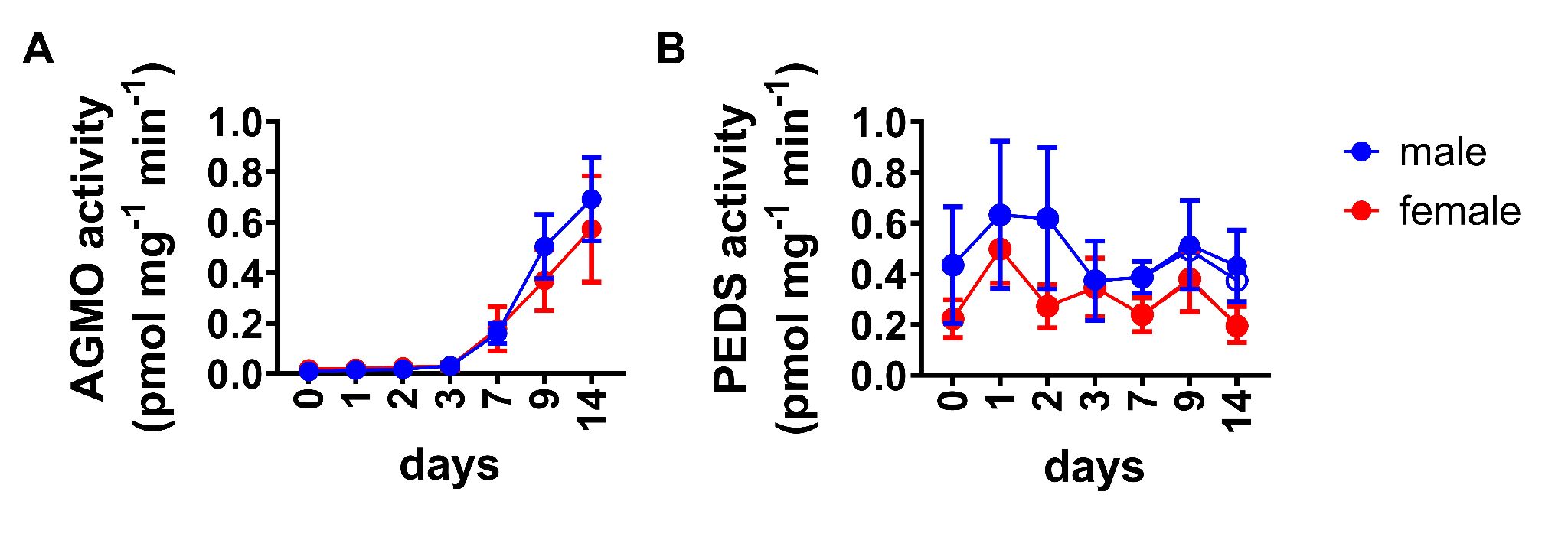


**Figure S1 Sex-specific analysis of AGMO and PEDS1 activity during *in vitro* adipocyte differentiation of human ASC.** Plots show **A)** analysis of AGMO enzyme activity during *in vitro* differentiation of male (blue) and female (red) human ASC (male: n = 10; female: n = 10) and **B)** PEDS1 enzyme activity during *in vitro* differentiation of male and female human ASC (male: n = 5; female: n = 7).

**
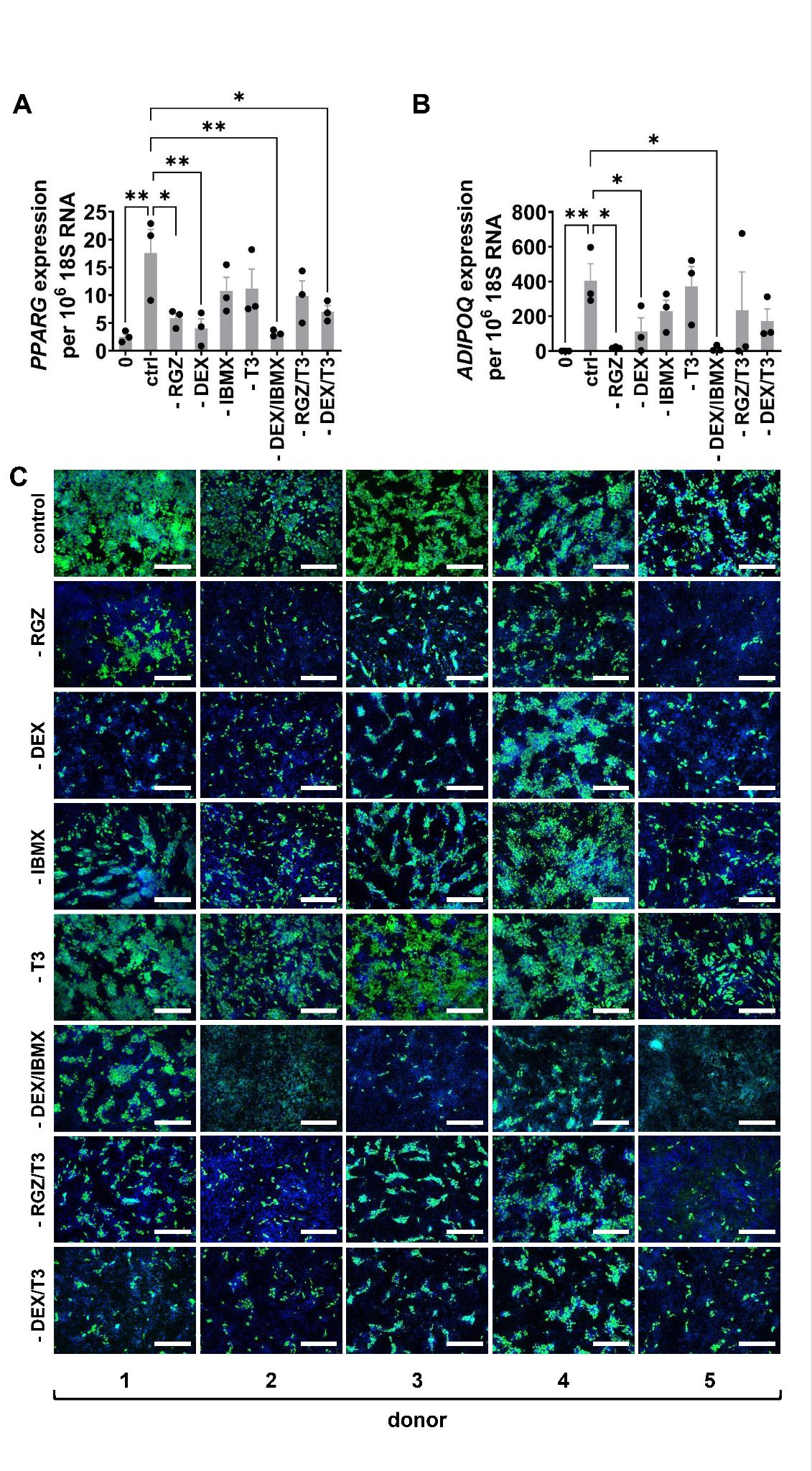
**

**Figure S2 Effects of omitting hormonal components on adipocyte-specific markers and lipid droplet formation.** Gene expression analysis of **A)** *PPARG* and **B)** *ADIPOQ* at day 14 of adipocyte differentiation with selected treatment regimens compared to undifferentiated cells at day 0 is depicted. Data is shown as mean ± SEM, n = 3. **C)** Representative pictures of Bodipy/Hoechst from each donor (n = 5) are shown. Scale bar = 500 µm. “-” indicates omission of DEX = dexamethasone, IBMX = 3-isobutyl-1-methylxanthine, RGZ = rosiglitazone and T3 = triiodothyronine as well as their combinations. Ctrl = cells exposed to the standard adipocyte supplemented with all hormones.


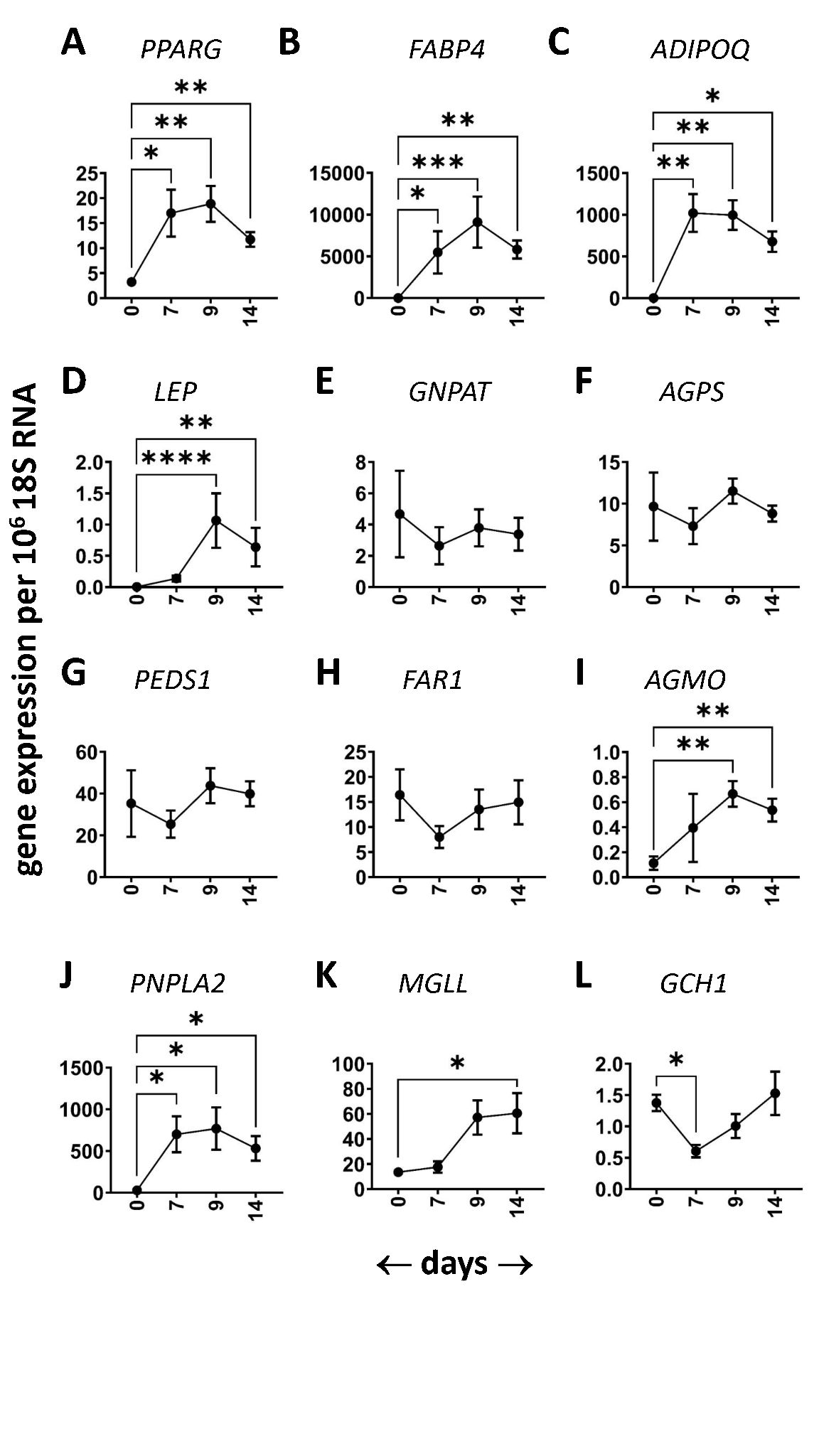


**Figure S3 Gene expression signatures of differentiation and lipid pathways during *in vitro* adipocyte conversion of human ASC.** Gene expression analysis was performed by RT-qPCR using Taqman technology. Expression of adipocyte markers **A)** *PPARG*, **B)** *FABP4*, **C)** *ADIPOQ* and **D)** leptin (*LEP*). Gene expression analysis of lipogenic genes such as **E)** *GNPAT*, **F)** *AGPS*, **G)** *PEDS1* and **H)** *FAR1*. Gene expression analysis of lipolytic genes **I)** *AGMO*, **J)** *PNPLA2* and **D)** *MGLL*. Expression of the rate limiting enzyme for tetrahydrobiopterin synthesis **L)** *GCH1* was additionally analyzed. Data is shown as mean ± SEM (n = 4-6).

**
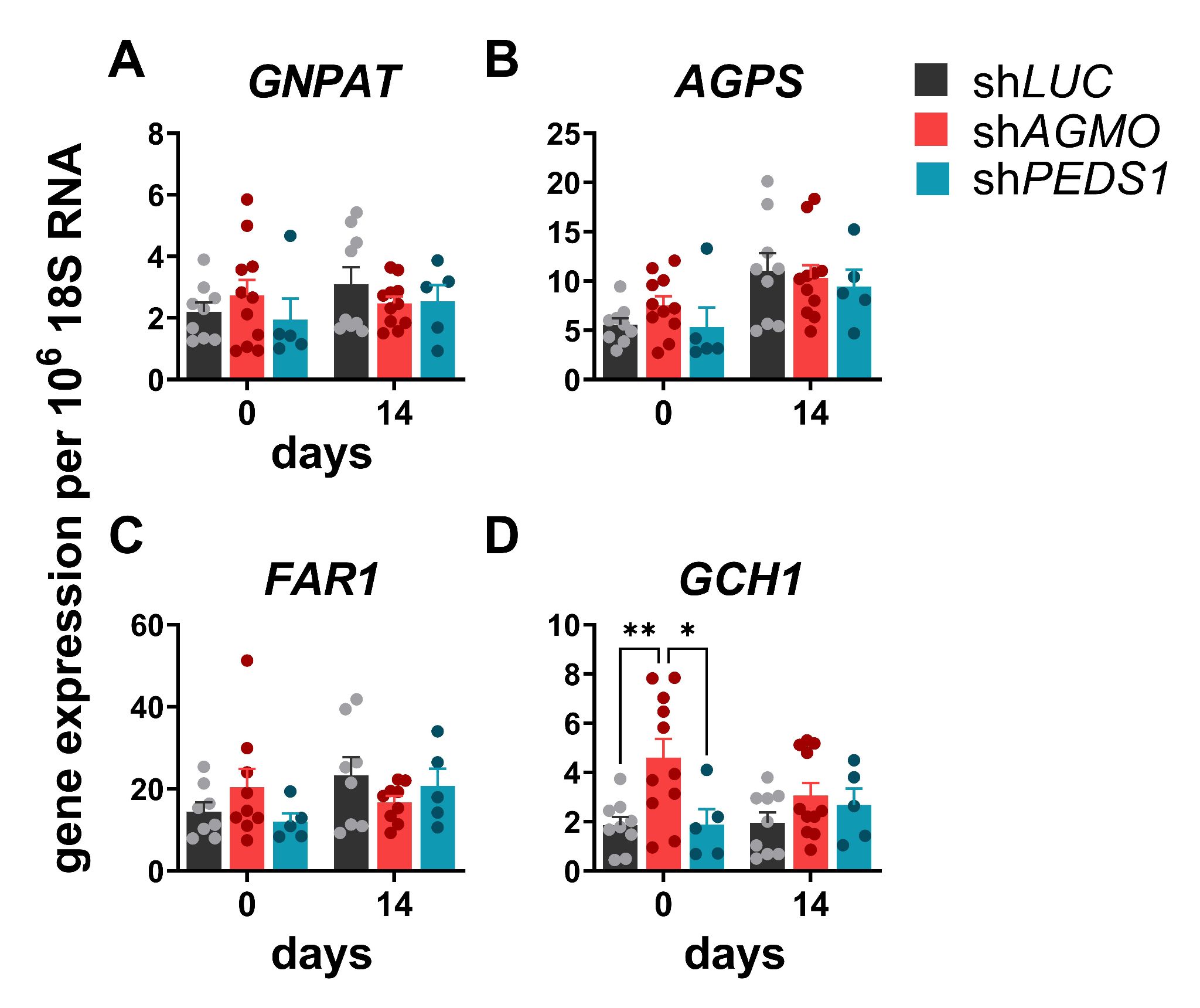
**

**Figure S4 Impact of *AGMO* and *PEDS1* KD on gene expression profiles during *in vitro* differentiation of human ASC.**

Expression of ether lipid metabolism genes **A)** *GNPAT*, *AGPS*, **C)** *FAR1* and **D)**  *GCH1*, involved in tetrahydrobiopterin biosynthesis, an essential cofactor for AGMO, are shown. sh*LUC*: black bars, n = 8 different donors; sh*AGMO*: red bars, n = 11 (for two different knockdown constructs using 8 different donors); sh*PEDS1*: blue bars, n = 5 different donors. Data is presented as mean ± SEM.


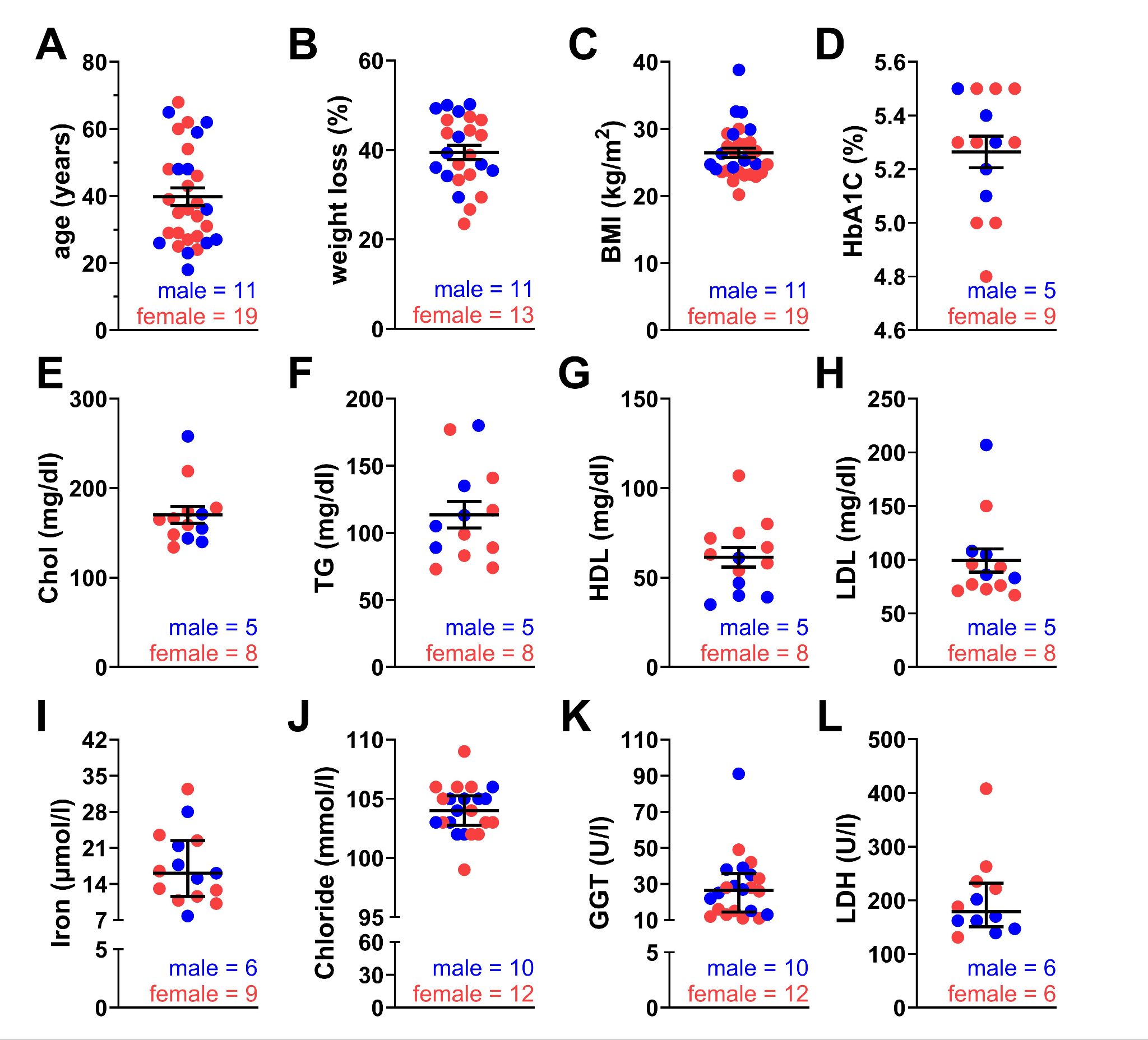


**Figure S5 Distribution of selected physiological cohort parameters related to either AGMO activity or *PEDS1* expression.**

Scatterplots showing the distribution of weight loss, BMI, age and basic blood parameters that were correlated with either AGMO activity or *PEDS1* expression. Samples were taken on average 51 days prior to surgery (max. time = 275 days and min. = 1 day). **A)** Age, **B)** weight loss until time of surgery and **C)** BMI. **D)** Hemoglobin A1C (HbA1C), **E)** cholesterol (Chol), **F)** triacylglycerol (TG), **G)** high density lipoprotein (HDL), **H)** low density lipoprotein (LDL), **I)** iron, **J)** chloride, **K)** GGT and **L)** lactate dehydrogenase (LDH).


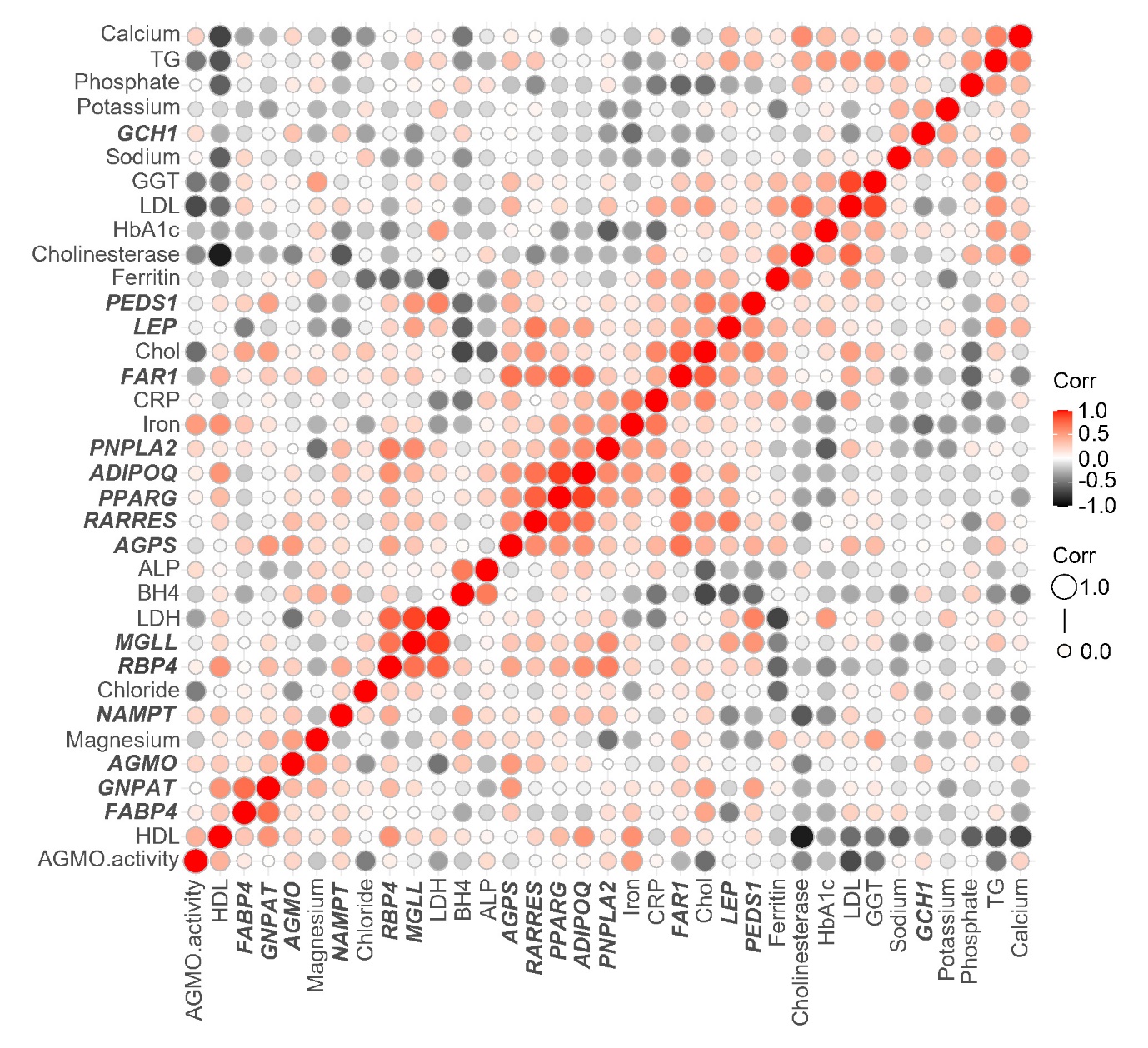


**Figure S6 Correlation matrix of all measured parameters in *in vivo* differentiated adipocytes.** Spearman correlation matrix of data obtained from human *in vivo* differentiated adipocytes computed in R. The scale, which is colored from red (positive correlation) to black (negative correlation), shows the absolute value of the corresponding correlation coefficients. The size of the circles indicates the strength of each correlation. Data was ordered using hclust for hierarchical clustering. All gene names are indicated with bold font for better visualization. Correlation coefficients and p-values and LFDR corrected p-values are given in Supplemental Material 2.

**
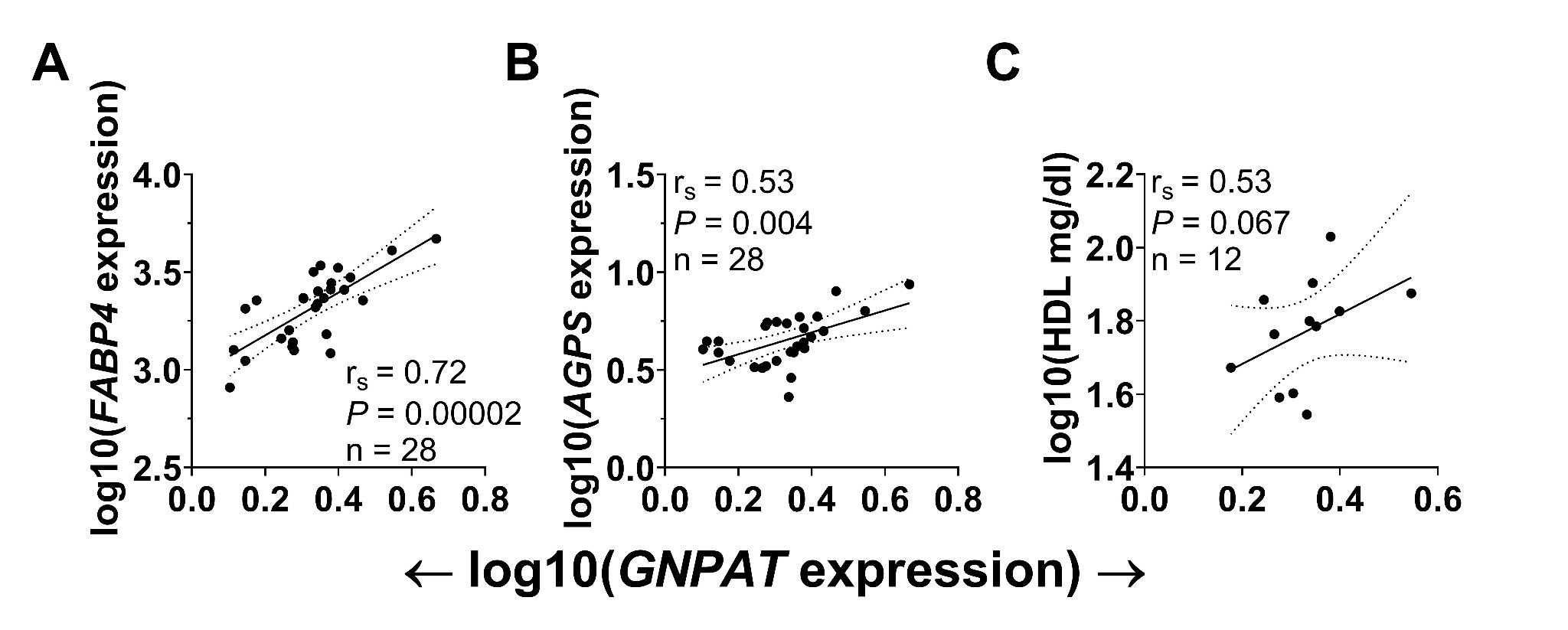
**

**Figure S7 Significant correlations of *GNPAT* gene expression with basic blood parameters and expression of analyzed genes in *in vivo* differentiated adipocytes.**

Spearman correlation analysis of *GNPAT* gene expression profiles in *in vivo* differentiated adipocytes with **A)** *FABP4* gene expression, **B)** *AGPS* gene expression and **C)** HDL is depicted. P-values shown are not adjusted. LFDR corrected p-values are given in Supplemental Material 2.


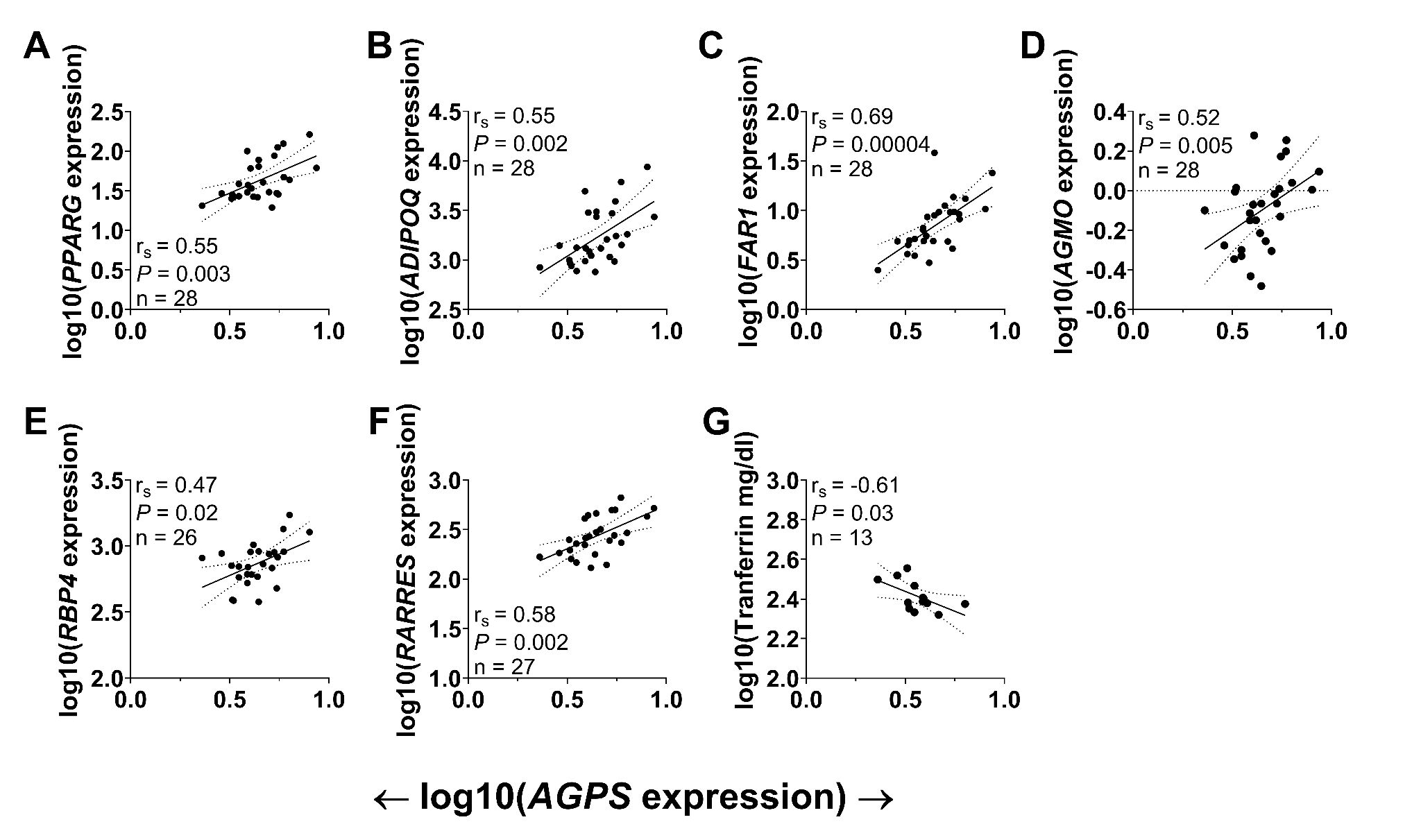


**Figure S8 Significant correlations of *AGPS* gene expression with basic blood parameters and expression of analyzed genes in *in vivo* differentiated adipocytes.**

Plots show Spearman correlation analysis of *AGPS* gene expression profiles in *in vivo* differentiated adipocytes with **A)** *PPARG* gene expression, **B)** *ADIPOQ* gene expression and **C)** *FAR1* gene expression, **D)** *AGMO* gene expression, **E)** *RBP4*, **F)** *RARRES* and **G)** transferrin. P-values shown are not adjusted. LFDR corrected p-values are given in Supplemental Material 2.


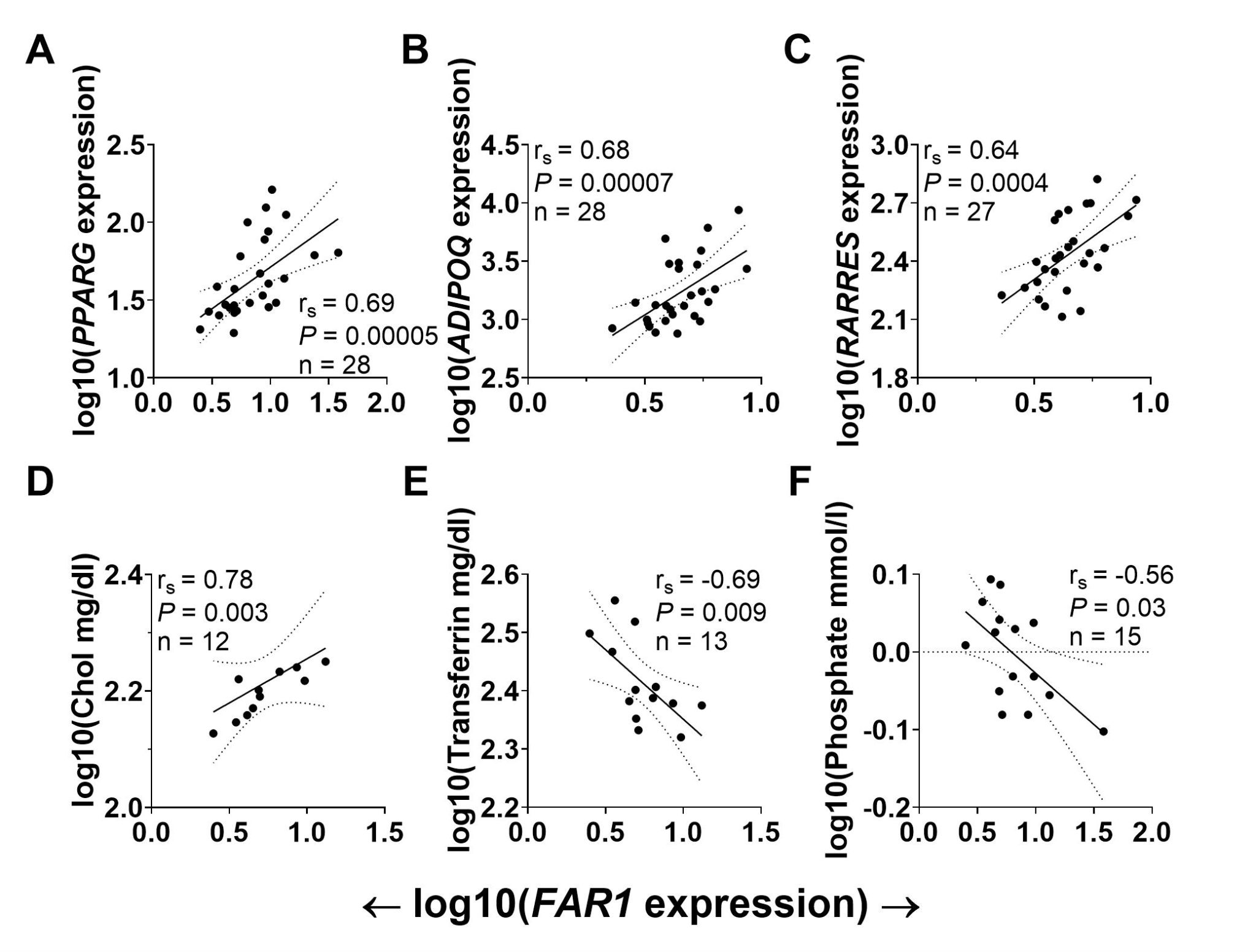


**Figure S9 Significant correlations of *FAR1* gene expression with basic blood parameters and expression of analyzed genes in *in vivo* differentiated adipocytes.**

Spearman correlation analysis of *FAR1* gene expression profiles in *in vivo* differentiated adipocytes with **A)** *PPARG* gene expression, **B)** *ADIPOQ* gene expression and **C)** *RARRES*, **D)** Chol, **E)** transferrin and **F)** phosphate. P-values shown are not adjusted. LFDR corrected p-values are given in Supplemental Material 2.


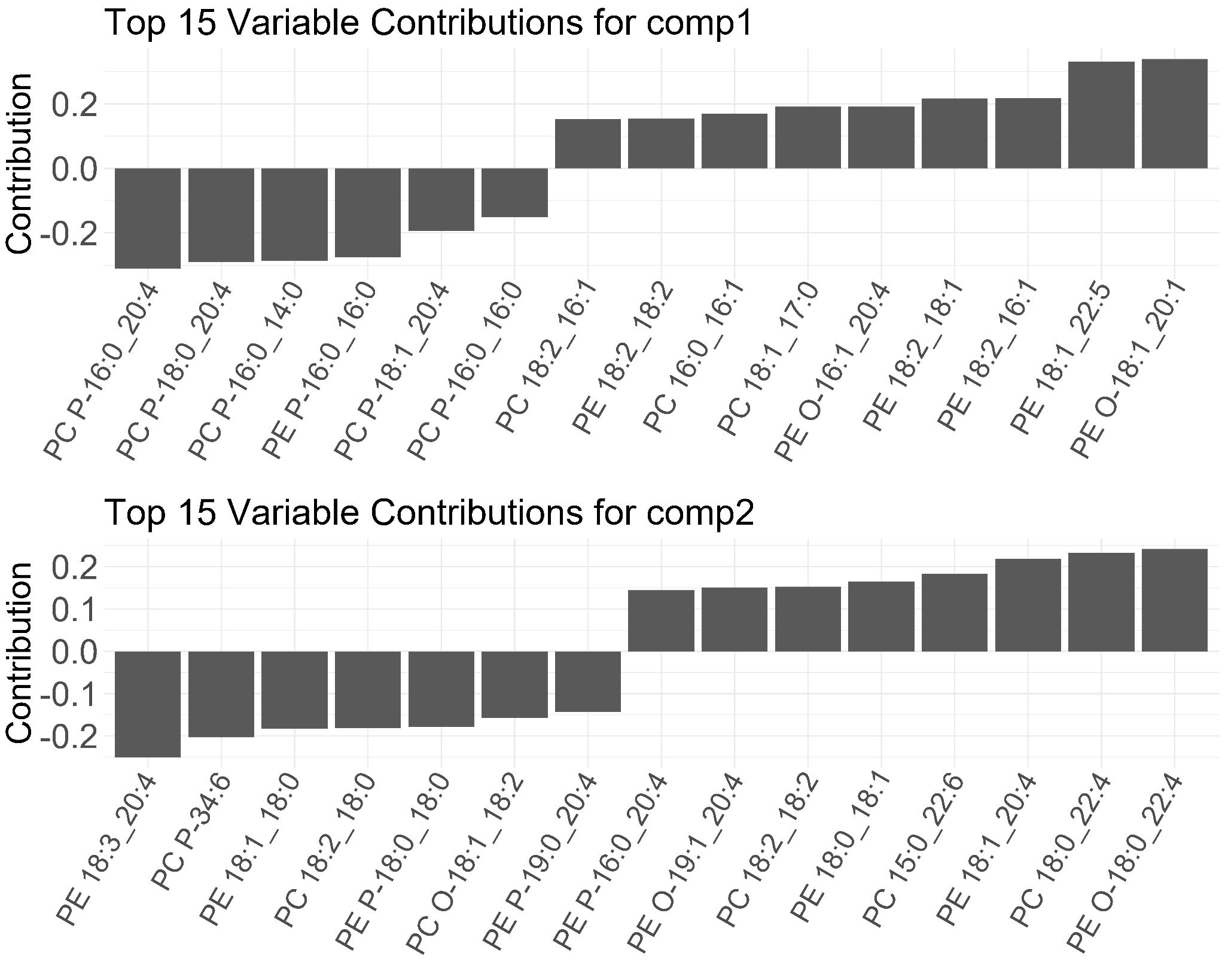


**Figure S10 Top 15 variable contributions to components 1 and 2 in sPLS-DA of lipidomics data.**

The bar plots represent the contributions of individual variables to each component. Variables are ranked based on their magnitude of contribution, with the top 15 contributions selected for clarity. Positive contributions indicate a direct association with the respective component, while negative contributions reflect an inverse relationship.

**
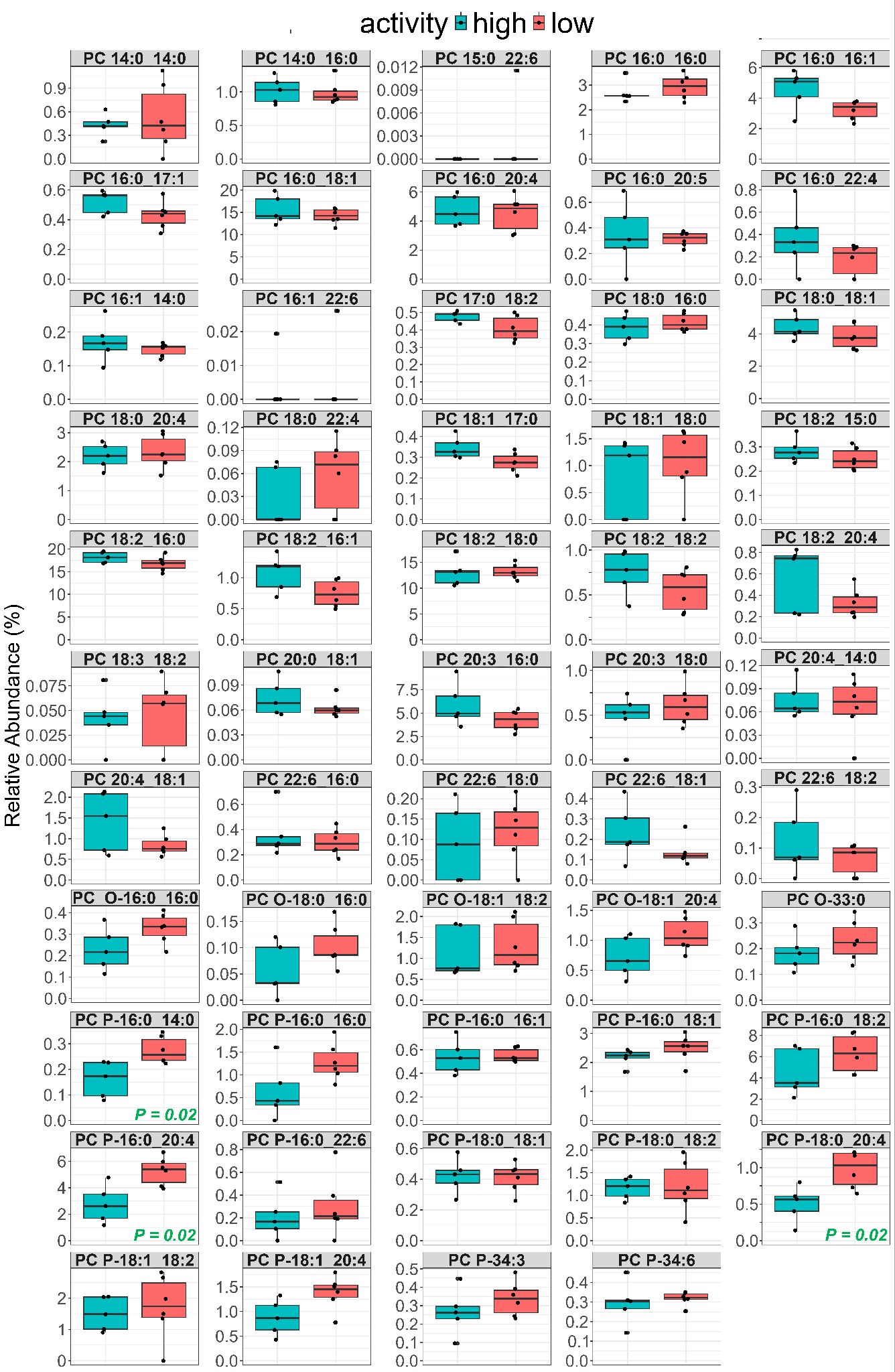
**

**Figure S11 PC lipid composition of high and low AGMO activity adipocytes.**

Boxplots showing the percent abundance of single lipid species of total PC. The molecular side chain composition is only shown where MS^2^ spectra were available. PC = phosphatidylcholine, PC(O) = plasmanyl phosphatidylcholine and PC(P) = plasmenyl phosphatidylcholine. Boxplots show median ± IQR (blue = AGMO activity high and red = AGMO activity low). Whiskers extend from minimum to maximum. Data was acquired by LC-MS/MS using DDA-PASEF. P values were added in green to significantly different lipid species. Primary adipocytes with high and low AGMO enzyme activities from 5 – 6 different donors.


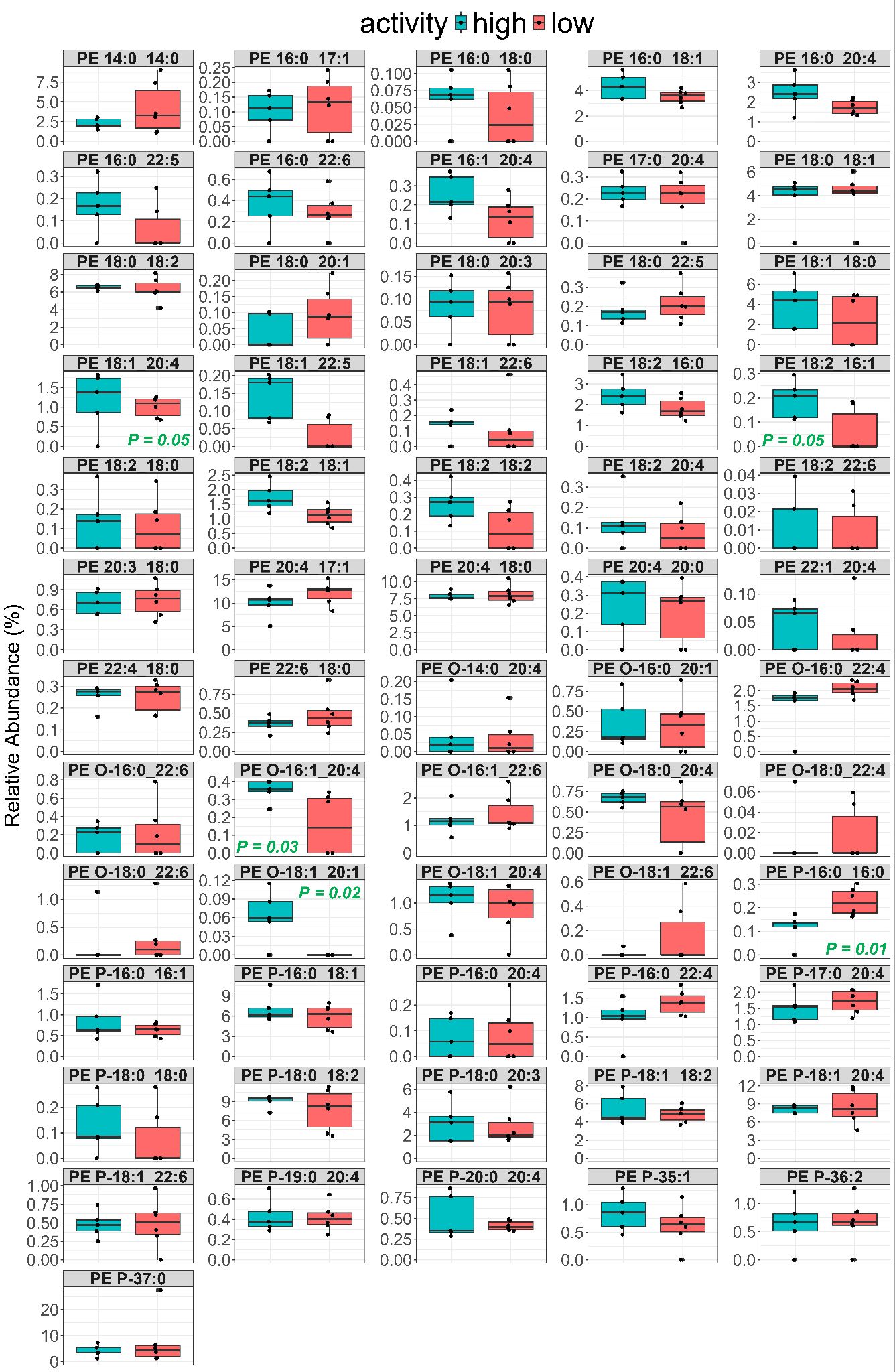


**Figure S12 PE lipid composition of high and low AGMO activity adipocytes.**

Boxplots showing the percent abundance of single lipid species of total PE. The molecular side chain composition is only shown where MS^2^ spectra were available. PE = phosphatidylethanolamine, PE(O) = plasmanyl phosphatidylethanolamine and PE(P) = plasmenyl phosphatidylethanolamine. Boxplots show median ± IQR (blue = AGMO activity high and red = AGMO activity low). Whiskers extend from minimum to maximum. Data was acquired by LC-MS/MS using DDA-PASEF. P values were added in green to significantly different lipid species. Primary adipocytes with high and low AGMO enzyme activities from 5 – 6 different donors.


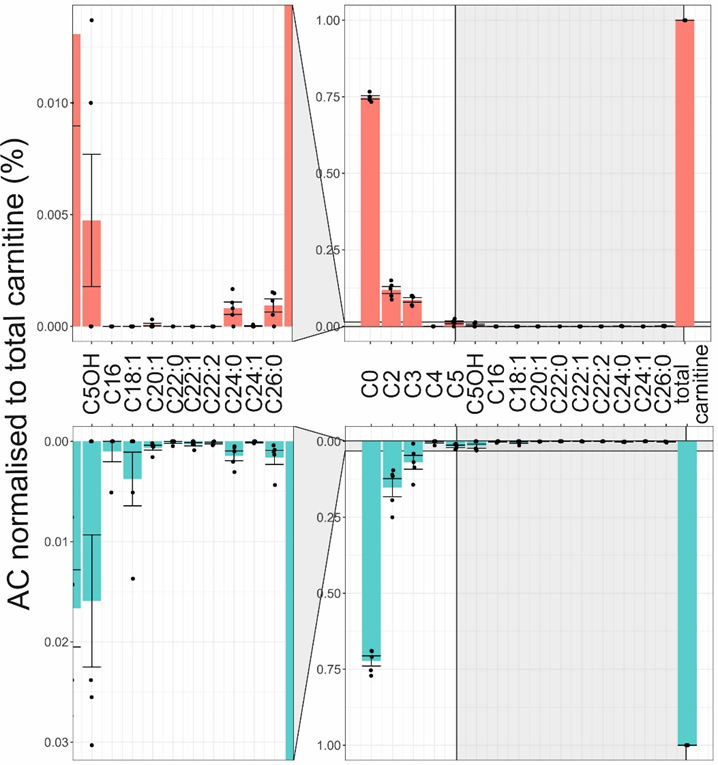


**Figure S13 Acylcarnitine species analysis for assessing differences in beta-oxidation by low or high AGMO enzyme activities**

Acylcarnitine species were normalised to total carnitine for comparison. Red bars show *in vivo* differentiated adipocytes with low AGMO activity. Blue bars show adipocytes containing high AGMO activity. Data is presented as mean ± SEM. Primary adipocytes with high and low AGMO enzyme activities from 5 – 6 different donors.
